# HSPB7 prevents cardiac conduction system defect through maintaining intercalated disc integrity

**DOI:** 10.1371/journal.pgen.1006984

**Published:** 2017-08-21

**Authors:** Wern-Chir Liao, Liang-Yi Juo, Yen-Ling Shih, Yen-Hui Chen, Yu-Ting Yan

**Affiliations:** 1 Institute of Biochemistry and Molecular Biology, National Yang-Ming University, Taipei, Taiwan; 2 Institute of Biomedical Science, Academia Sinica, Taipei, Taiwan; The Jackson Laboratory, UNITED STATES

## Abstract

HSPB7 is a member of the small heat-shock protein (HSPB) family and is expressed in the cardiomyocytes from cardiogenesis onwards. A dramatic increase in HSPB7 is detected in the heart and blood plasma immediately after myocardial infarction. Additionally, several single-nucleotide polymorphisms of HSPB7 have been identified to be associated with heart failure caused by cardiomyopathy in human patients. Although a recent study has shown that HSPB7 is required for maintaining myofiber structure in skeletal muscle, its molecular and physiological functions in the heart remain unclear. In the present study, we generated a cardiac-specific inducible HSPB7 knockout mouse and demonstrated that the loss of HSPB7 in cardiomyocytes results in rapid heart failure and sudden death. The electrocardiogram showed cardiac arrhythmia with abnormal conduction in the HSPB7 mutant mice before death. In HSPB7 CKO cardiomyocytes, no significant defect was detected in the organization of contractile proteins in sarcomeres, but a severe structural disruption was observed in the intercalated discs. The expression of connexin 43, a gap-junction protein located at the intercalated discs, was downregulated in HSPB7 knockout cardiomyocytes. Mislocalization of desmoplakin, and N-cadherin, the intercalated disc proteins, was also observed in the HSPB7 CKO hearts. Furthermore, filamin C, the interaction protein of HSPB7, was upregulated and aggregated in HSPB7 mutant cardiomyocytes. In conclusion, our findings characterize HSPB7 as an intercalated disc protein and suggest it has an essential role in maintaining intercalated disc integrity and conduction function in the adult heart.

## Author summary

The intercalated disc is an indispensable structure that connects neighboring cardiomyocytes. It is also considered to be a single functional unit for cellular electric, mechanical, and signaling communication to maintain cardiomyocyte rigidity and synchrony. Mutation or defect in intercalated disc components usually results in distortions in the structure of intercalated discs and lethal cardiac abnormalities in patients. In this study, we found that the dynamic expression and subcellular location of HSPB7 are highly associated with intercalated disc component protein, N-cadherin, during the assembly and maturation of intercalated discs in cardiomyocytes. To identify the functional role of HSPB7 in the adult heart, we conducted a loss-of-function study of HSPB7 using a gene conditional knockout approach. We found that the loss of HSPB7 quickly results in the disruption of the intercalated disc structure, decreasing the expression of connexin 43 and mislocalization of N-cadherin and desmoplakin, and further inducing arrhythmic sudden death. In conclusion, our mouse model demonstrates that HSPB7 is required to maintain the structure and function of gap-junction complexes and intercalated discs, which has important implications for human heart disease.

## Introduction

HSPB7, also known as cardiovascular heat-shock protein (cvHsp), is a member of the small heat-shock protein (sHSP or HSPB in mammals) family that shares a conserved α-crystallin domain in the C-terminal region [1, 2]. The sHSPs characteristically function as ATP-independent molecular chaperones assisting intracellular protein assembly and cytoskeleton formation under normal conditions and suppressing the aggregation of denaturing proteins in resistance to stress [3]. Many sHSPs are expressed in cardiac and skeletal muscles [4, 5] and the phenotypes of their mutations are seen in muscle diseases. For example, mutation in HSPB5 was found to induce desmin-related skeletal muscle myopathy and cardiomyopathy [6, 7]. Previous studies have also demonstrated that HSPB2, HSPB5, and HSPB6 can protect against myocardial ischemia-reperfusion injury and suppress pressure overload cardiac hypertrophy [8–12]. Consequently, these studies suggest that sHSPs are crucial in protecting striated muscles from damage caused by stress and injury.

HSPB7 is the most highly expressed sHSP gene in the heart [1]. Increasing expression of HSPB7 can be detected in the monocrotaline-induced hypertrophic right ventricle [1], aging skeletal muscle [13], and dystrophin-deficient MDX diaphragm in mice [14]. Despite being the most potent suppressor of sHSP against the aggregation of the mutated huntingtin protein [15], HSPB7 was reported to be a potential early biomarker of myocardial infarction and an independent risk factor for acute coronary syndrome [16]. Overexpression of HSPB7 can reduce the amount of tachypacing-induced F-actin stress fibers through attenuation of the RhoA-GTPase pathway [17]. Recent studies have reported that an intronic single-nucleotide polymorphism (SNP) rs1739843 in HSPB7 is highly associated with heart failure (HF) [18–20], dilated cardiomyopathy (DCM) [21], and idiopathic DCM [22] in human patients. Although rs1739743 and another 11 additional HSPB7 SNPs were further confirmed to be associated with heart failure, the additional SNPs were also found to be intronic or synonymous. As such, HSPB7 SNPs are predicted to have no effect on its protein sequence or function, suggesting a role as a marker for the position of a genetically linked functional variant located outside HSPB7 [23]. However, all the previous findings still imply the role of HSPB7 in cardiac pathogenesis. Recently, the studies conducted using gene knockdown in zebrafish demonstrated that HSPB7 is required for the formation of the left-right axis and cardiac morphogenesis [24, 25]. Although these findings demonstrate the essential role of HSPB7 in cardiac development and functional maintenance, the functions of HSPB7 in adult heart still remain unclear.

Using an HSPB7 inducible-conditional knockout (CKO) model approach, here we demonstrate that the loss of HSPB7 leads to cardiomyopathy and arrhythmic sudden death. Lack of HSPB7 causes the disruption of the intercalated disc (ID) structure, resulting in abnormal localization of ID component proteins and defect of the cardiac conduction function. Furthermore, we found that filamin C (FLNC), the interaction protein of HSPB7, was mislocalized and aggregated in HSPB7 CKO cardiomyocytes. Thus, our results suggest that HSPB7 acts as a novel cardiac ID protein to maintain the structural integrity of the ID and the cardiac functions of the adult heart.

## Results

### HSPB7 is localized at intercalated discs and adjacent to the Z-line of the adult cardiac muscle

**Fig 1:**
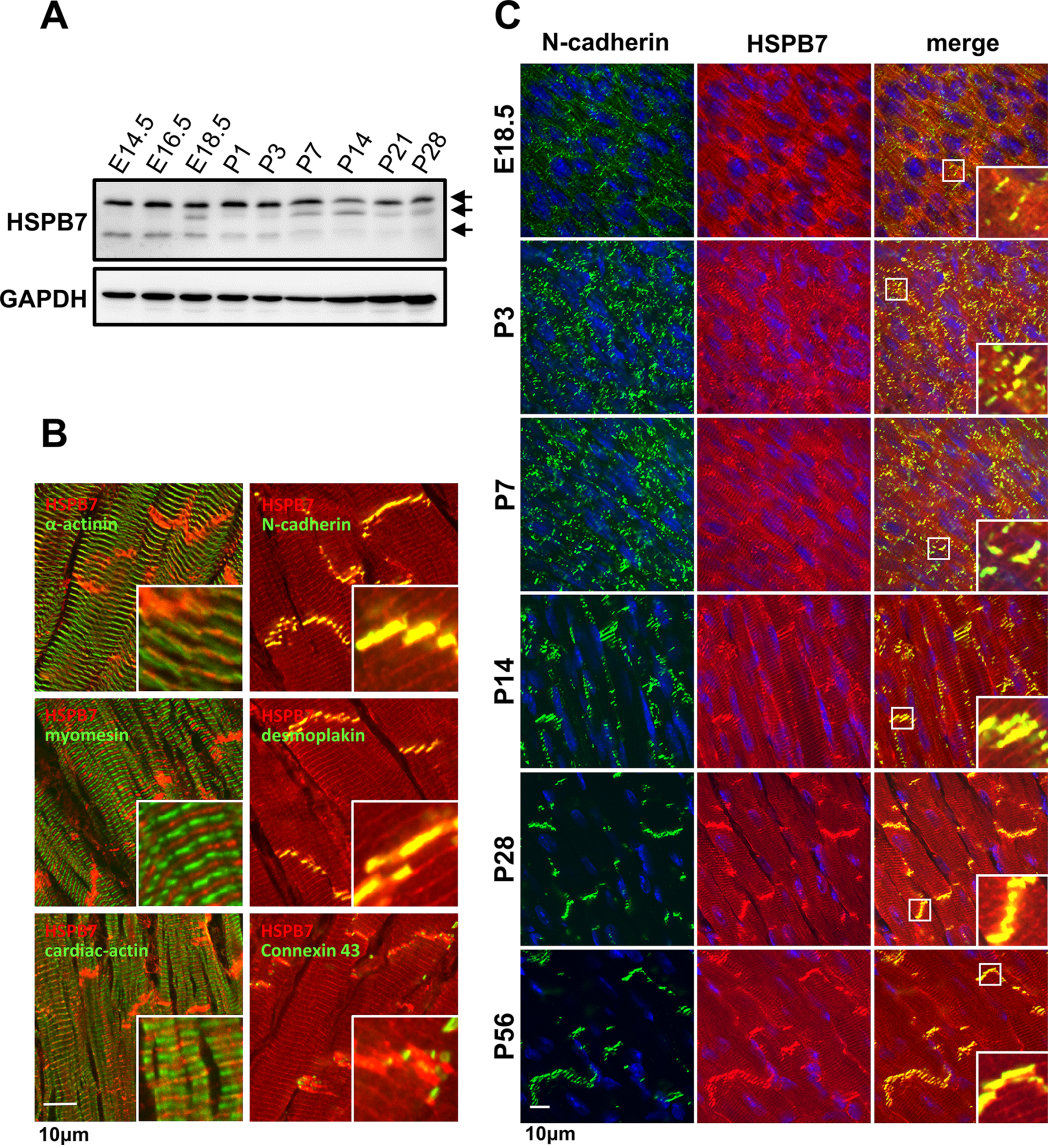
Expression and localization of HSPB7 in cardiac muscle. (A) Immunoblot analysis of the cardiac muscle showing HSPB7 constitutive expression from the embryonic stages to adulthood (E14.5 to P28) with multiple forms of HSPB7 at different molecular masses (arrows). (B) Subcellular localization of HSPB7 in the cardiac muscle of adult mice. The heart sections were stained with antibodies against HSPB7 (red) and desmoplakin (desmosome), α-actinin (Z-line), myomesin (M-line), N-cadherin (adhering junction), connexin 43 (gap junction), and cardiac-actin (I-bend). HSPB7 mainly localizes at the intercalated discs and is adjacent to the Z-line with a striated pattern. (C) Colocalization of HSPB7 with N-cadherin during development. Heart sections from the embryonic stages to adulthood (E14.5 to P56) were stained with antibodies against HSPB7 (red) and N-cadherin (green). The nucleus was visualized through Hoechst 33342 staining. Insets show the representative areas with higher magnification. Scale bar: 10 μm.

To explore the functional role of HSPB7 in the heart, we first analyzed the expression of HSPB7 in the heart. Immunoblot analysis revealed that HSPB7 is expressed in the heart from embryonic day 14.5 (E14.5) to postnatal day 28 (P28) (Fig 1A) in multiple forms with different molecular masses (arrows in Fig 1A). The subcellular localization of HSPB7 was determined by confocal fluorescence microscopy in longitudinal sections of the adult mouse heart. Double labeling with sarcomere markers, α-actinin as the Z-line, myomesin as the M-line, and cardiac-actin as the I-band showed that HSPB7 is expressed adjacent to the Z-line (Fig 1B). Localization of HSPB7 at the IDs was verified by co-staining with N-cadherin (adherens junction), desmoplakin (desmosomes), and connexin 43 antibody (gap junction; Fig 1B). HSPB7 is present as a diffusion pattern in bands of regular periodicity, and is not completely colocalized with N-cadherin at E18.5 (Fig 1C). After birth, bright patches of HSPB7 staining were observed at P3 and appear to have a compact pattern of adherens junction localization at P14, which is indicated by colocalization with N-cadherin. In the adult heart, HSPB7 staining was seen as faint striations, mostly concentrated at the ends of cardiomyocytes. Our results indicate that HSPB7 is highly colocalized with N-cadherin during the assembly and maturation of IDs, suggesting that HSPB7 may be involved in organizing and maintaining the cardiac cytoarchitecture.

### HSPB7 CKO mice develop lethality with cardiomyopathy

**Fig 2:**
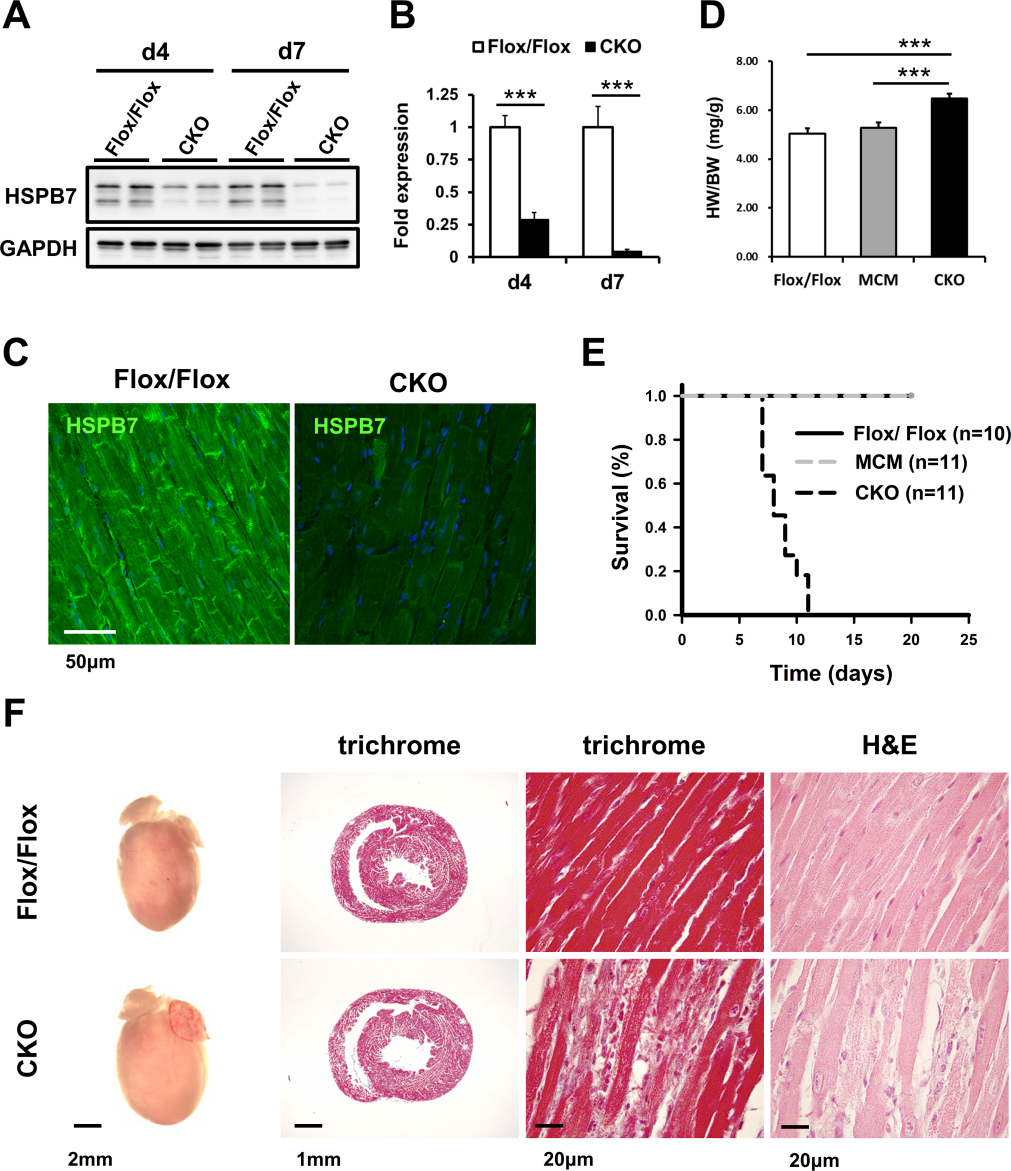
Characterization of HSPB7 CKO mouse. (A) Immunoblot analysis for HSPB7 protein expression levels in control (Flox/Flox) and CKO animals 4 days (d4) and 7 days (d7) after tamoxifen administration. The GAPDH signal shows the loading of samples between lanes. (B) Quantitative analysis for immunoblot analysis of HSPB7 expression protein levels in cardiac tissue from control and CKO mice. Seven days after the first tamoxifen administration, HSPB7 protein expression dropped to less than 10% in CKO animals, as determined by immunoblot blot (HSPB7 compared with GAPDH). Data are presented as means ± SD. (C) Double staining of the left ventricle section with HSPB7 and Hoechst 33342 in the control and CKO hearts at d7 after tamoxifen administration. HSPB7 was no longer present at the intercalated disc and sarcomere. Scale bar: 20 μm (D) Significant increase in heart weight in HSPB7 CKO mice (n = 8; *p = 0.00027). (E) Kaplan–Meier survival curve of HSPB7 CKO and control mice. (F) Representative whole mounts (left), Masson’s trichrome (middle left and right), and hematoxylin-eosin–stained (right) transverse sections of control and CKO mouse hearts. Histological analysis showed inflammatory infiltration in the myocardium, identified as mostly lymphocytes and plasma cells, in HSPB7 CKO hearts at d7 after tamoxifen administration. In addition, Masson’s trichrome staining showed no significant collagen deposition in HSPB7 CKO hearts, as compared to control mice.

**Fig 3:**
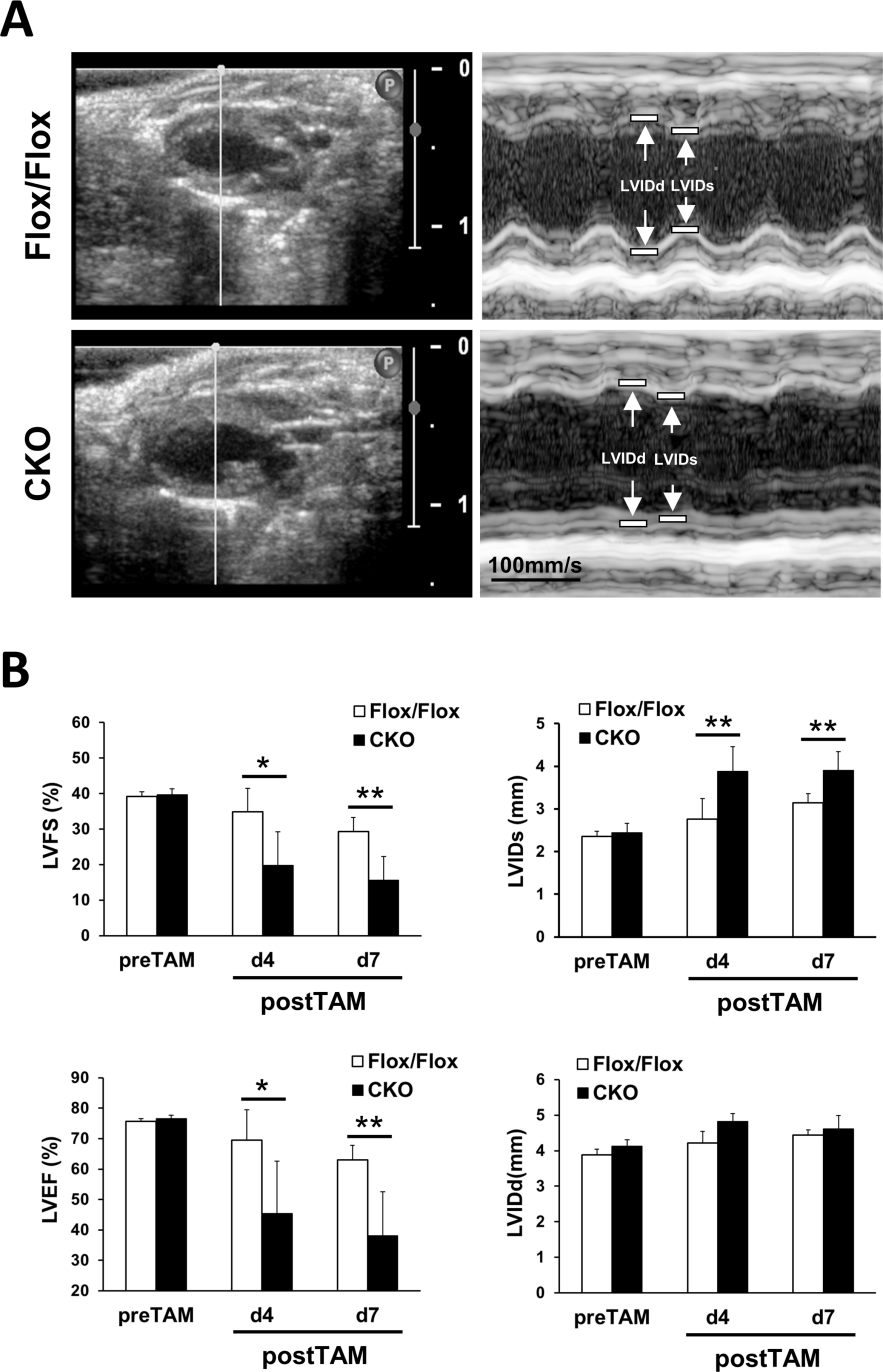
Echocardiographic measurements of HSPB7 CKO and control mice. (A) Representative two-dimensional and M-mode echocardiographic images of the HSPB7 CKO and control hearts. (B) Echocardiographic analysis in the HSPB7 CKO and control mice before and at 4 days (d4) and 7 days (d7) after tamoxifen administration. Left ventricular ejection fraction (LVEF), left ventricular fractional shortening (LVFS), left ventricular end-systolic internal diameter (LVIDs), and left ventricular end-diastolic internal diameter (LVIDd). Data are means ± SD; n = 5 per group. *, *P* < 0.05 **, *P* < 0.01 relative to the control.

To elucidate the function of HSPB7 in the heart, a tamoxifen-induced CKO mouse line (MCM/HSPB7^Flox/Flox^) was established using HSPB7^Flox/Flox^ intercrossed with MCM mice [26]. HSPB7 CKO and littermates (8- to 10-week-old) including HSPB7^Flox/Flox^ and MCM mice were administered with tamoxifen for four consecutive days. Immunoblot analysis of heart lysates revealed a 75% and 95% reduction in HSPB7 protein levels in the hearts of CKO mice compared with their control HSPB7^Flox/Flox^ littermates (n = 4; Fig 2A and 2B) at d4 and d7 after tamoxifen administration, respectively. Immunofluorescence staining and confocal microscopy also revealed a dramatic decrease in HSPB7 at the intercalated discs and sarcomeres at d7 after tamoxifen administration in CKO mice (Fig 2C). The CKO mice displayed a moderate increase in heart weight/body weight ratio compared with their HSPB7^Flox/Flox^ littermates (n = 8; Fig 2D). Notably, we found that ablation of HSPB7 in the cardiomyocytes led to rapid mouse death within 12 days (n = 11). By contrast, tamoxifen-treated HSPB7^Flox/Flox^ (n = 10) or MCM control mice (n = 11) appeared healthy throughout the course of tamoxifen treatment (Fig 2E). Histological analysis showed slight inflammatory infiltrate, cardiomyocyte disarray, and enlarged myocytes with hyperchromatic nuclei in the CKO myocardium. However, fibrosis was not detected in the mutant heart by using Masson’s trichrome stain (Fig 2F). To further explore the functional pathological phenotype of the HSPB7 CKO mice in more detail, we then analyzed cardiac function through echocardiography. Echocardiographic analysis showed that the contractility of the CKO mice was significantly impaired compared with the control mice. We observed a significant reduction in LV fractional shortening (%FS; 29.3% ± 4.0 for HSPB7^Flox/Flox^ versus 15.6% ± 6.7 for CKO) and ejection fraction (%EF; 63.0% ± 4.8 for HSPB7^Flox/Flox^ versus 38.1% ± 14.5 for CKO) in CKO mutants compared with controls (n = 5; Fig 3) at d7 after tamoxifen administration. These results imply that ablation of HSPB7 in the myocardium leads to cardiomyopathy, HF, and sudden death.

### Abnormal cardiac conduction properties and sudden arrhythmic death in HSPB7 CKO mice

**Fig 4:**
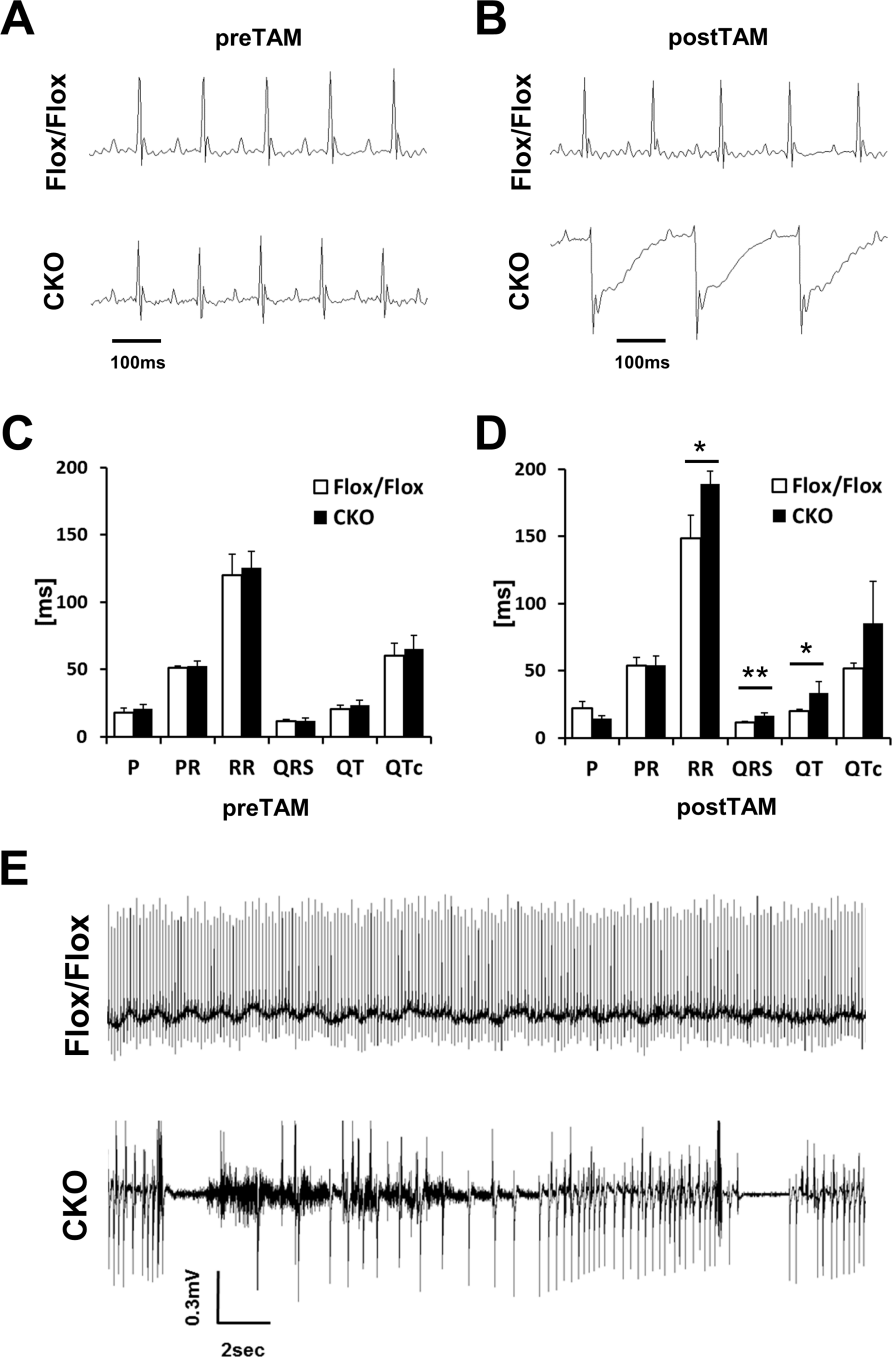
Electrical conduction is impaired in HSPB7 CKO hearts. (A and B) Annotated ECG curve of the HSPB7 CKO and control animals before (A) and 7 days after (B) the first tamoxifen injection. (C and D) Quantification of the ECG changes in HSPB7 CKO and control animals before (C) and 7 days after (D) the first tamoxifen administration. N = 5 per group. Data are presented as means ± SD. *, *P* < 0.05 **, *P* < 0.01 relative to the control. (E) Representative telemetric 2-lead ECG recording of a tamoxifen-treated HSPB7 CKO and control mice. Telemetry ECG recordings of lethal arrhythmias in HSPB7 CKO mice. n = 2 per group.

Given the cardiomyopathy and sudden death observed in HSPB7 CKO mice, we speculated that the normal electrophysiological activities were disturbed in the HSPB7 mutant hearts. For routine monitoring of cardiac activity during anesthesia, we used the ECG, which revealed an abnormality in the HSPB7 CKO hearts (Fig 4A–4D). ECG measurements in 8-week-old mice revealed no difference in QRS duration, QT interval, or PQ interval between HSPB7^Flox/Flox^ and MCM/HSPB7^Flox/Flox^ mice (Fig 4A and 4C). By d7 after tamoxifen administration, although the conduction of the electrical activity from the atrium to ventricle of the CKO mice was normal (PR interval) (53.6 ± 6 ms vs. 54.3 ± 6 ms; Fig 4B and 4D), the depolarization and repolarization of the CKO ventricle were disturbed. This is reflected in the prolonged QRS complex (11.4 ± 0.8 ms vs. 16.4 ± 2.1 ms) and QT interval (20.0 ± 1.26 ms vs. 33.3 ± 8.5 ms) after induction of the CKO (Fig 4B and 4D). To further investigate the nature of sudden cardiac death and evaluate possible underlying ECG abnormalities, miniaturized telemetric transmitter devices were implanted in 8- to 10-week-old MCM, HSPB7^Flox/Flox^, and HSPB7^Flox/Flox^ mice to record their cardiac rhythm [27] (Fig 4E). We recorded the time interval for 2 hours before and at d4, d7, and d14 after tamoxifen administration to HSPB7 CKO and control mice. The HSPB7 CKO mice were continuously recorded at d7 after tamoxifen administration until death. All the control mice exhibited normal sinus rhythms, with no evidence of ventricular ectopy. We also observed ST segment abnormalities in HSPB7 CKO mice similar to the result of surface ECG recordings at d7 after tamoxifen administration (S1 Fig). In the HSPB7 CKO mice during the continuous recording period, we captured the abrupt onset of spontaneous ventricular tachyarrhythmia, confirming that the death was arrhythmic.

### Structural defects of IDs and sarcomeres in HSPB7 CKO cardiomyocytes

**Fig 5:**
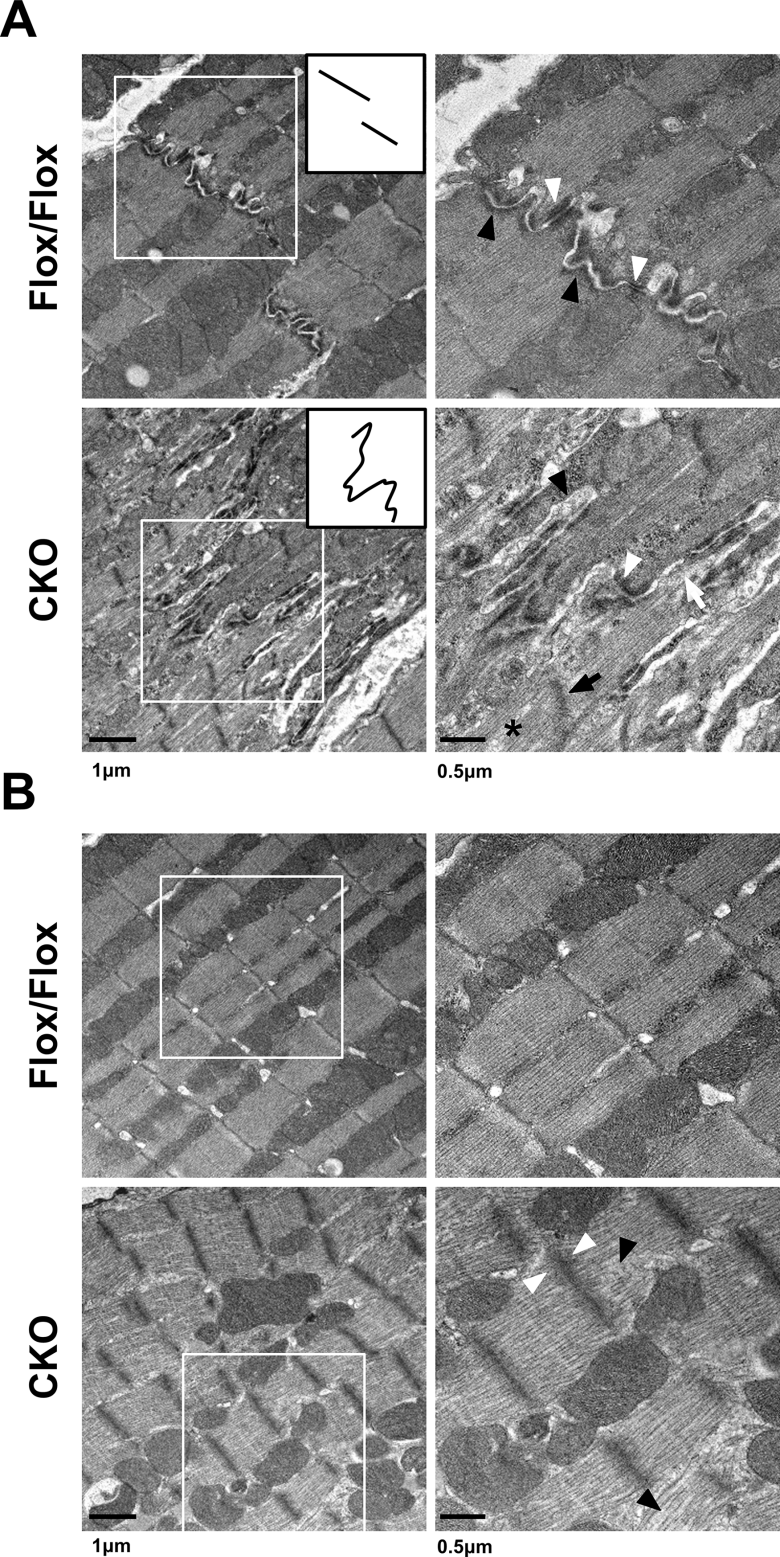
Ultrastructural study of control and HSPB7 CKO hearts. Transmission electron micrographs (TEMs) of ventricular myocardium from HSPB7 CKO and control mice at d7 after tamoxifen administration. Right panels are higher-magnification views of the boxed areas in the left panels. (A) Normal intercalated disc structures were visible in the control hearts. The inset provides a simplistic representation of the morphology of the intercalated discs. Higher-magnification images showed abnormal adherens junctions (black arrowheads) and desmosomes (white arrowheads) with widened gaps of the intercalated discs in HSPB7 mutant hearts. Abnormal Z-line (black arrow), filament disruption (asterisk), and detachment of myofibrils at the intercalated disc (white arrow) were also observed in the CKO mice. (B) The ultrastructure of the sarcomeres at the center part of the cardiomyocyte was slightly distorted. Higher-magnification images showed (right panel) loose actin filaments (black arrowhead) and wider, less dense Z-lines (white arrowhead) compared with the controls. n = 3 per group. Scale bar: 250 nm.

To examine the myofibril organization and cell–cell contacts at the ultrastructural level, transmission electron micrograph (TEM) analysis was performed on control and CKO hearts (Fig 5) at d7 after tamoxifen administration. Intercalated disc structures were visible in the HSPB7^Flox/Flox^ hearts, with clear adherens junctions and desmosomes represented by submembranous electron dense material adjacent to the intercellular space between the myocytes. By contrast, the structures of the mutant IDs were highly convoluted and disorganized (Fig 5A). Higher-magnification images revealed abnormal adherens junctions (black arrowhead in Fig 5A) and desmosomes (white arrowhead in Fig 5A) with widened gaps at the IDs of HSPB7 mutant cardiomyocytes. The ultrastructure of the sarcomeres at the center part of the mutant cardiomyocytes was slightly distorted, showing loose actin filaments (black arrowhead in Fig 5B) and wider, less dense Z-lines (white arrowhead in Fig 5B) compared with the controls. The TEM images of the sarcomeres proximal to the intercalated discs showed an abnormal Z-line (black arrow in Fig 5A), disrupting filaments (asterisk in Fig 5A), and lacunae spaces at the sites of myofibril attachment at the IDs (white arrows in Fig 5A). These sarcomere defects in the HSPB7 CKO myocardium presumably reflect the lack of myofibril anchorage at the plasma membrane.

### Disrupted localization of ID component proteins in HSPB7 CKO cardiomyocytes

**Fig 6:**
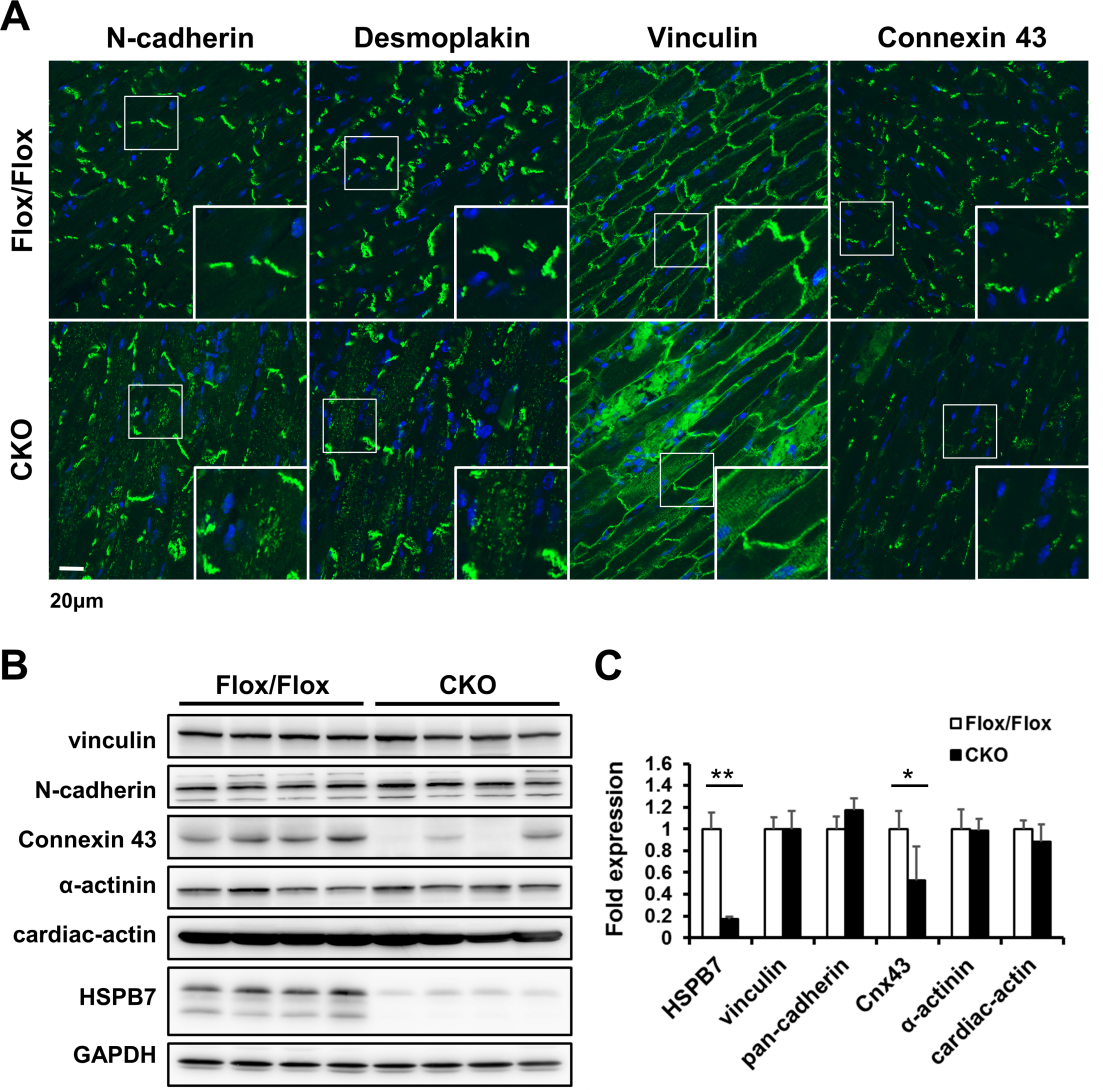
Expression and localization of ID complex proteins in HSPB7 CKO hearts. (A) Confocal micrographs of longitudinal sections of the cardiac muscle of control and CKO mice at d7 after tamoxifen administration. Specific antibodies were used to identify the distributions of intercalated disc components: N-cadherin, desmoplakin, vinculin, and connexin 43. In HSPB7 CKO hearts, the staining of desmoplakin and N-cadherin were distributed throughout the cytoplasm, but little was observed in control hearts. Connexin 43 was absent from the intercalated discs in HSPB7 CKO hearts. The insets show representative areas at a higher magnification. The nucleus was visualized through Hoechst 33342 staining. n = 4 per group. Scale bar: 20 μm. (B) Immunoblotting of intercalated discs and sarcomeric-associated proteins in HSPB7 CKO and control hearts. GAPDH signal shows the loading of the samples between the lanes. n = 4 per group. (C) Quantitative analysis of immunoblot analysis of protein levels in cardiac tissue from control and CKO mice. Seven days after the first tamoxifen injections, connexin 43 protein expression dropped to < 40% in CKO animals, as determined by immunoblot analysis. Data are presented as means ± SD. *, *P* < 0.05, **, *P* < 0.01 relative to control.

Because HSPB7 is expressed at both the IDs and adjacent to the Z-line in the adult heart, we next examined whether the loss of HSPB7 affects the cell–cell junctions of the IDs and the sarcomeric apparatus. The components of the ID, desmoplakin (a cytoplasmic desmosomal protein), N-cadherin (an adherens junction protein) and connexin 43 (a gap junction protein) were examined through immunofluorescence staining and confocal microscopy (Fig 6A). In HSPB7 CKO hearts, the cytoplasmic localization of desmoplakin and N-cadherin were observed in the HSPB7 CKO heart (Fig 6A) but not in the HSPB7^Flox/Flox^ control heart. Moreover, depletion of HSPB7 in the IDs resulted in a significant decrease in connexin 43 in the myocardium (Fig 6A). Immunoblot blot analysis further confirmed a reduction in connexin 43 in the HSPB7 CKO heart compared with the HSPB7^Flox/Flox^ control heart (Fig 6B and 6C), whereas N-cadherin and desmoplakin levels remained unchanged in the mutant hearts (Fig 6B and 6C). Furthermore, the expression pattern of vinculin (a costamere marker) was characterized relatively normally (Fig 6A) in HSPB7 CKO cardiomyocytes. Confocal microscopy revealed the normally striated structure of the Z-line (α-actinin) and the M-line (myomesin) (S2 Fig), consistent with the results of the TEM. Likewise, immunoblot analysis also showed the same expression levels of sarcomeric protein (Fig 6B and 6C) in the HSPB7 CKO hearts. Taken together, these data support the notion that HSPB7 is required to maintain the ID structure including the components of gap junctions, desmosomes, and adherens junctions.

### Loss of HSPB7 results in FLNC aggregation and disruption of membrane integrity

**Fig 7:**
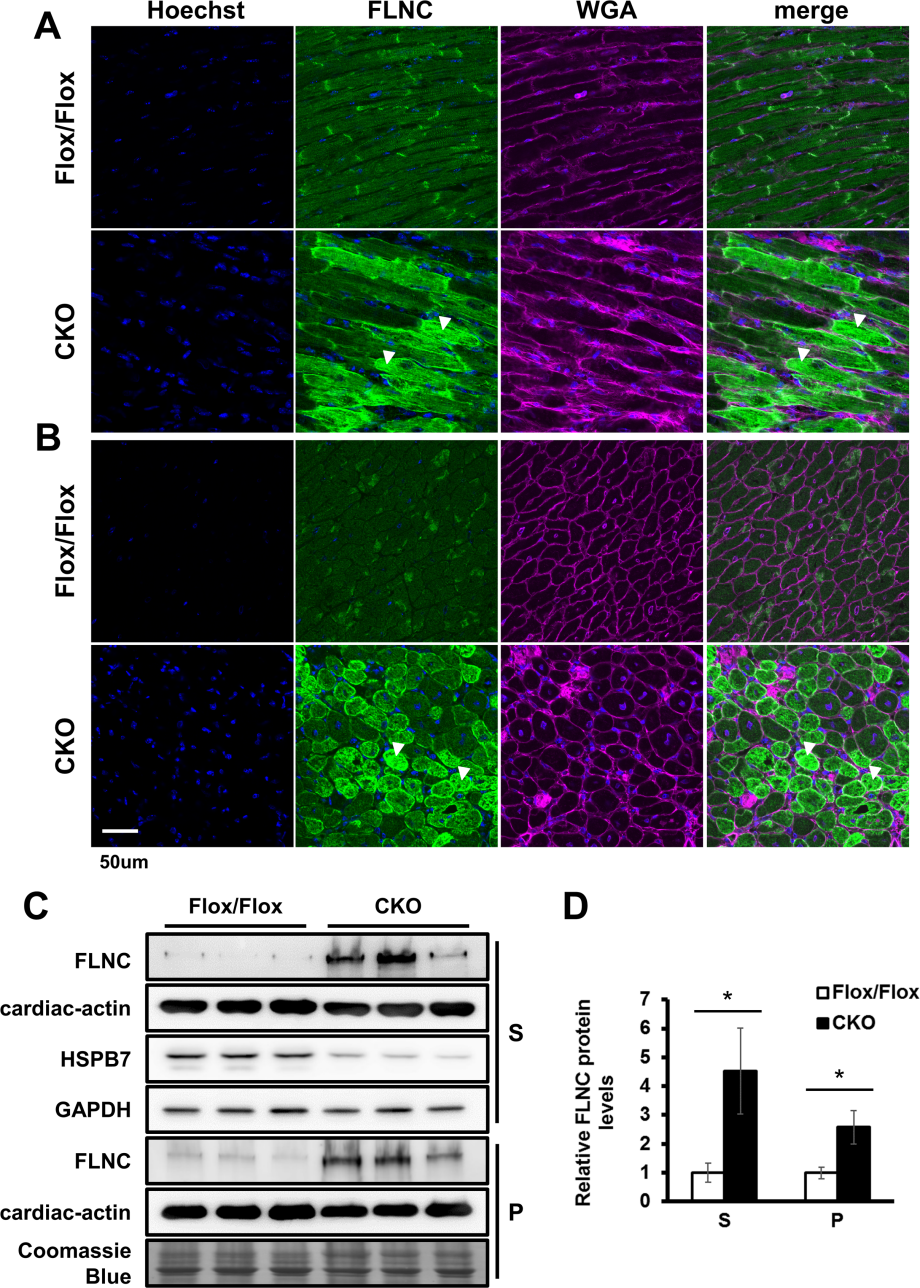
Loss of HSPB7 results in FLNC protein upregulation and aggregation. Confocal micrographs of longitudinal sections (A) and cross-sections (B) of the heart. FLNC aggregation (arrowheads) was observed only in the mutant cardiomyocytes. Extracellular matrix accumulation was labeled using wheat germ agglutinin (WGA). The nucleus was visualized through Hoechst 33342 staining. Scale bar: 50 μm. (C) Immunoblot analysis and quantitation (D) of the expression levels of FLNC in the cardiac muscle. The muscle homogenate supernatant (S) and pellet (P) fractions were analyzed from control and CKO mice at d7 after tamoxifen administration. GAPDH and Coomassie Blue staining were used to verify the loading amount in the supernatant and pellet. n = 3 per group. Data are presented as means ± SD. *, *P* < 0.05 relative to the control.

To further identify the possible functional target(s) affected by HSPB7 loss, we examined the interactions of ID component proteins with HSPB7 by co-immunoprecipitation. Interestingly, only FLNC (but not N-cadherin, desmoplakin, or connexin 43) was found to interact with HSPB7 in heart lysate (S3 Fig). FLNC has been found to be the interaction protein of HSPB7, and the loss of HSPB7 in skeletal muscles can cause progressive myopathy with FLNC aggregation [28]. The confocal microscopy showed that HSPB7 mainly colocalized with FLNC adjacent to the Z-line and at IDs in the cardiomyocytes (S4 Fig). Furthermore, a significant increase in FLNC expression was detected in the HSPB7 CKO heart at d7 after tamoxifen administration (Fig 7A and 7B) and is consistent with the immunoblotting analysis in the supernatant and pellet fractions of heart lysates showing the upregulation of FLNC protein expression (Fig 7C and 7D). Furthermore, along with the FLNC aggregation detected in the myocardium (arrowheads in Fig 7A), an accumulation of extracellular matrix (WGA staining in Fig 7A and 7B) was also observed in the myocardium of the HSPB7 CKO mice.

To understand whether the aggregation of FLNC is the cause of the disruption of ID structure, immunoblotting analysis was performed in HSPB7 CKO mice at d4 after tamoxifen administration (S5 Fig). The results showed that the downregulation of connexin 43 occurred before the upregulation of FLNC in the HSPB7 CKO heart. These results suggest that the reduction of connexin 43 may not be caused by the overexpression or aggregation of FLNC. Additionally, double staining of FLNC with desmoplakin or N-cadherin showed that the mislocalization of desmoplakin or N-cadherin (arrowheads in S6 Fig) and FLNC aggregation (arrows in S6 Fig) did not always occur at the same cardiomyocytes of the HSPB7 CKO heart at d7. Our findings indicate that the overexpression or aggregation of FLNC protein may not be the direct cause for the disruption of ID structure in HSPB7 CKO cardiomyocytes. We next examined whether cell integrity was affected by the loss of HSPB7 in the heart [29, 30], since the sarcolemma disruption was observed in the HSPB7 skeletal muscle specific CKO mouse. Using EBD uptake analysis, we found that the HSPB7 CKO heart presented blue coloration compared with the control. Consistent with our previous study [28], a high uptake of EBD (red) was detected in the HSPB7 mutant cardiomyocytes (S7 Fig).

### Adenovirus-mediated expression of Cre in HSPB7^Flox/Flox^ mouse hearts

**Fig 8:**
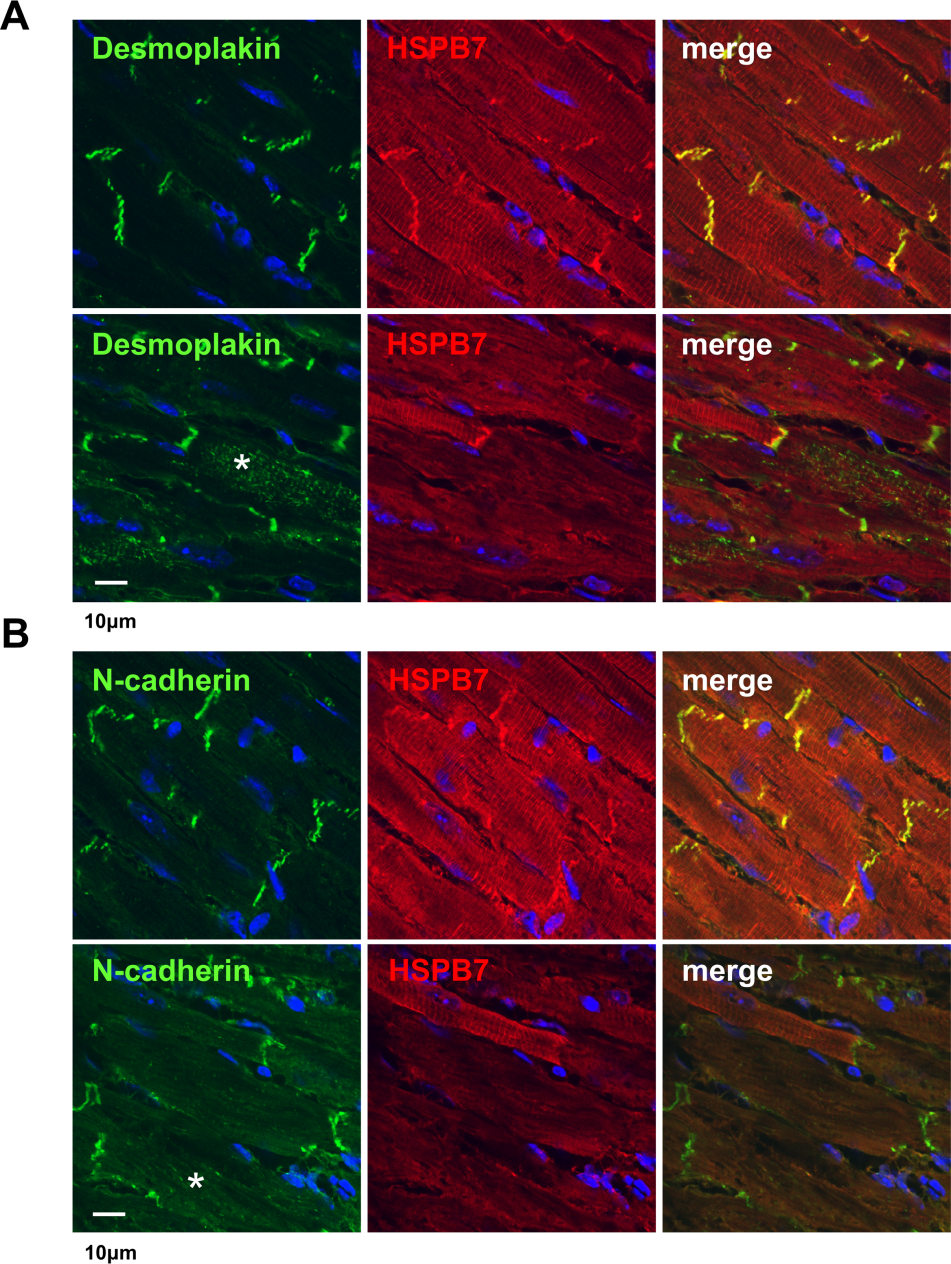
Intramyocardial injection with Adeno-Cre in HSPB7^Flox/Flox^ mice. Confocal micrographs of the cardiac muscle of the HSPB7^Flox/Flox^ mice 8 days after intramyocardial injection with Adeno-Cre virus. n = 3 per group. The heart sections were co-immunostained with antibody against HSPB7 (red) and specific antibodies (green) were used to identify the distributions of intercalated disc components: desmoplakin (A) and N-cadherin (B). (A) In HSPB7^Flox/Flox^ hearts, desmoplakin was distributed throughout the cytoplasm (asterisk in lower panel) at the HSPB7 depleted region compared with the control region (upper panel). (B) N-cadherin exhibited partial misexpression at the HSPB7 depleted region (asterisk in lower panel) compared with the control region (upper panel). The nucleus was visualized through Hoechst 33342 staining (blue). Scale bar: 10 μm.

Tamoxifen in αMHC-MerCreMer mice could induce a DNA damage response leading to HF and death [31]. To exclude the possibility that the phenotype of HSPB7 CKO resulted from tamoxifen toxicity, we performed gene elimination through direct intramyocardial injection with the Adeno-Cre virus in HSPB7^Flox/Flox^ mice. The expression patterns of ID components and FLNC were evaluated at d8 after Adeno-Cre virus injection by immunofluorescence staining. Adeno-Cre virus treatment efficiently reduced HSPB7 expression at the injected region of the heart (the upper Cre region shown in S8 Fig). As shown in the immunofluorescence analysis, HSPB7 was eliminated, and a decrease in expression of connexin 43 was observed in the HSPB7-depleted regions of the Adeno-Cre virus-injected heart. Additionally, high magnification confocal images of these regions showed that the mislocalization of desmoplakin (Fig 8A) and N-cadherin (Fig 8B) occurred in HSPB7-depleted cardiomyocytes. Consistent with the results for the HSPB7 CKO heart, confocal fluorescence microscopy revealed that the immunoreactivity of FLNC significantly increased in HSPB7 depleted regions of the Adeno-Cre virus-injected heart. Overall, our results indicate a direct functional and cell-autonomous role for HSPB7 in maintaining FLNC stability and ID structure in cardiomyocytes.

## Discussion

In this study, we have shown that HSPB7 plays an essential role in maintaining ID integrity to prevent cardiac arrhythmogenic failure. We demonstrated the dynamic expression and subcellular location of HSPB7 in cardiac muscle from the embryonic stage to adulthood. We found that HSPB7 is highly colocalized with N-cadherin during the assembly and maturation of IDs. Importantly, we demonstrated that the ablation of HSPB7 in adult mouse hearts leads to (i) the disruption of ID structure with distorted expression and location of ID components, (ii) defects in myofibrillar organization and membrane integrity in cardiomyocytes, and (iii) the development of abnormal conductive activity with arrhythmic sudden death.

The IDs are an indispensable structure that connect neighboring cardiomyocytes, which is essential for electric, mechanical, and signaling communication between adjacent cells [32]. Loss of ID components, such as plakoglobin [30] and N-cadherin [33] causes cardiac arrhythmic death with distortion of ID structures and downregulation of ID protein in mice. A previous study demonstrated that dysregulation and mislocalization of cadherin may dissipate the contractile force across the plasma membrane leading to impaired force transmission and dilated cardiomyopathy [34]. Consistent with the arrhythmogenic lethal phenotype, HSPB7 CKO mice exhibited severe disruption of the ID structure with mislocalization of N-cadherin and desmoplakin in the cardiomyocyte cytosol. Likewise, we speculate that loss of HSPB7 may cause the structural instability of ID components and further affect the localization and function of N-cadherin and desmoplakin.

Furthermore, several genetic mutations of desmoplakin [35] and plakoglobin [36] have been found to cause arrhythmogenic cardiomyopathy (AC) in humans. AC is a complex disorder and is considered to be a progressive disease of the IDs with clinical manifestations, including progressive loss of cardiomyocytes, inflammatory infiltrates, and compensatory replacement with fibrofatty tissue, leading to HF, severe ventricular tachyarrhythmias, and sudden cardiac death. HSPB7 CKO mice do not present with the typical phenotype seen in AC patients and lacks severe DCM and fibrofatty replacement phenotypes. It is possible that the mutant animals die too soon from sudden death (within 2 weeks) to observe a long-term compensation effect.

Adherens junctions and desmosomes are organized independently of gap junctions [37]. The loss of protein in either the adherens junctions or desmosomes can decrease the expression of gap junction proteins [38] and results in arrhythmogenesis development [33, 39]. The HSPB7 CKO mutant presents a much more severe phenotype compared with connexin 43 CKO and other adherens junctions or desmosome mutant mice [30, 33, 40], suggesting loss of HSPB7 could affect more than one of these protein functions. These results suggest that HSPB7 has a pivotal role as an ID protein for maintaining the structure and functional complexes of IDs.

The molecular mechanism by which HSPB7 maintains ID structure remains unclear. Our previous study identified FLNC, an actin-binding protein, as the HSPB7 interaction protein [28]. Loss of HSPB7 in skeletal muscle causes myofibrillar disorganization and sarcolemma disruption [28]. FLNC participates in the attachment of the sarcomere’s Z-lines to the costamere and IDs allowing cell-to-cell mechanical force transduction [41]. In the present study, myofibril organization appeared distorted in the mutant hearts that had wider, less dense Z-lines and loose actin filaments. Such a phenotype may have been caused by defects in the IDs. Ablation of ID proteins, such as N-cadherin [33] and plakoglobin [42], can lead to distortion of the sarcomere, which may reflect loss of myofibril tension because of a lack of myofibril anchorage at the plasma membrane. Alternatively, loss of FLNC can also result in extensive disruption of the thick and thin filaments and the loss of distinct Z-lines in mice [43]. Furthermore, the down regulation of connexin 43 is before the occurrence of FLNC up-regulation and aggregation (d4); and the mislocalization of desmoplakin or N-cadherin in HSPB7 CKO cells is not always detected with the aggregation of FLNC. Taken together, our results suggest that the up-regulation of FLNC may not be the first step in the resulting phenotype. Loss of HSPB7 would cause the structural instability of FLNC and then trigger the chaperone-assisted selective autophagy (CASA) pathway to increase the gene expression and aggregation of FLNC [28]. The CASA machinery can incorporate tension sensing, autophagosome formation, and transcription regulation to maintain filamin protein homeostasis in mammalian cells [44, 45]. Loss of HSPB7 would facilitate FLNC unfolding or conformational changes that would further activate the CASA pathway, thereby leading to FLNC upregulation and then aggregation. A recent study reported that 23 different truncating variants in FLNC are highly associated with variable fractures of DCM and AC [46]. The presence of FLNC aggregates has also been identified in cardiomyocytes of patients with cardiomyopathy [47] and cardiac arrhythmia [48]. However, our results indicated that loss of HSPB7 resulting in the mislocalization of desmoplakin or N-cadherin would not be directly affected by the effect of FLNC overexpression and aggregation. On the other hand, the possibility still cannot be ruled out that the interaction between HSPB7 and FLNC is required for maintaining IDs and myofibrillar functional structures in the adult heart.

In conclusion, our results provide the first comprehensive study characterizing HSPB7 as an ID protein and revealing information regarding the biological function of HSPB7 in the adult myocardium. The loss of HSPB7 results in the disruption of the ID structure with abnormal cardiac conduction function and thus induces arrhythmic sudden death, indicating the phenotype in the HSPB7 CKO mice is at least partly similar to that in human AC patients. Although it has not yet been reported, given the severity of the cardiac phenotype in our animal model, the functional mutation variants of HSPB7 gene could be identified in patients with AC. Thus, our mouse model demonstrates that HSPB7 is required for the structural integrity and function of gap-junction complexes and IDs, a finding which has vital implications for human heart disease.

## Materials and methods

### Ethics statement

All animal experiments were performed in accordance with the guidelines established by the Institutional Animal Care and Use Committee (IACUC) of Academia Sinica. All the experimental protocols were approved by IACUC and the approval number is 10–12–113. Mice were treated according to institutional protocols or perfused transcardially under deep Avertin anesthesia for further perfusion.

### Generation of HSPB7 CKO mice

To generate inducible cardiac-specific HSPB7 CKO mutants, transgenic mice expressing a tamoxifen-inducible Cre recombinase protein under the control of the α-myosin heavy chain promoter, αMHC/MerCreMer mice (MCM) [26], were intercrossed with HSPB7Flox/Flox mice [28] in a mixed 129S6/SvEvTac with a C57BL/6 genetic background. To induce Cre recombination, 8- to 10-week-old male MCM/HSPB7^Flox/Flox^ mice were treated with 40 mg/kg of tamoxifen (cat#L5647, Sigma) by intraperitoneal injection for 4 consecutive days. Tamoxifen was dissolved in corn oil at a concentration of 10 mg/mL heated to 37°C for 1 h. Mice were sacrificed at 4 and 7 days following the initiation of the tamoxifen treatment, and Cre-negative littermates were used as controls. All animal experiments were performed using protocols approved by the Institutional Animal Care and Use Committee, IBMS, Academia Sinica.

### Preparation of tissue extracts and immunoblotting

For tissue extracts, the hearts were dissected from anesthetized mice, rinsed with cold PBS, blotted dry, weighed, and then homogenized using TissueLyser LT (QIAGEN) in 1X Cell Lysis Buffer (cat#9803, Cell Signaling Technology) with a complete protease inhibitor cocktail (cat#11836145001, Roche Applied Sciences). For fractionation assay, mouse hearts were homogenized in lysis buffer containing 9M urea. The insoluble pellet fraction was sedimented through centrifugation at 16,000 g for 15 min. An equal volume of 2X SDSPAGE gel sample buffer was added to the supernatants and insoluble pellet fraction. After heating at 100°C for 10 min, the supernatants were stored at −80°C as aliquots until use. For western blotting experiments, protein extracts (30 μg of total protein) were separated on 8% or 15% polyacrylamide gels and blotted onto PVDF membranes (Millipore). Membranes were blocked with blocking buffer (5% nonfat dry milk, 10 mM of Tris–HCl, pH 7.6, 150 mM NaCl, and 0.1% Tween 20) and incubated with the primary antibody at 4°C overnight. After incubation with peroxidase-conjugated secondary antibodies, proteins were visualized using enhanced chemiluminescence reagents (Millipore) and detected using an ImageQuant LAS 4000 mini system (GE Healthcare Life Sciences). Densitometric analyses were performed using ImageJ software. The protein levels of GAPDH were used to normalize the results. The primary antibodies used included mouse monoclonal antibodies anti-α-actinin (clone EA-53, Sigma-Aldrich; 1:1000.), anti-Actin, cardiac (clone AC1-20.4.2, Sigma-Aldrich; 1:1000), anti-connexin43 (clone CXN-6, Sigma-Aldrich; 1:1000), anti-GAPDH (clone 6C5, Millipore Corporation; 1:3000); and anti-Vinculin (clone hVIN-1, Sigma-Aldrich, Inc.); rabbit polyclonal antibodies anti-FLAG (F7425, Sigma-Aldrich; 1:1000), antipan-cadherin (#C3678, Sigma-Aldrich; 1:1000); goat polyclonal antibodies anti-FLNC (K-18, Santa Cruz Biotechnology; 1:200); and guinea pig polyclonal antibody anti-HSPB7 (G11W, LTK BioLaboratories; 1:1000).

### Histopathology

Mouse heart tissues were collected, fixed with 10% formalin, buffered with phosphate, and embedded in paraffin. Tissue sections (5 μm) were subjected to hematoxylin and eosin and Masson’s trichrome staining using standard procedures [49].

### Confocal immunofluorescence analysis

For immunofluorescence staining, heart tissue was isolated from HSPB7 CKO mice, directly embedded in optimal cutting temperature compound (OCT), and cryosections (16-μm sectioned) were prepared. The sections were postfixed in 2% paraformaldehyde, blocked with 2% bovine serum albumin, and incubated with primary antibodies at 4°C overnight. The primary antibodies used included mouse monoclonal antibodies anti-α-actinin (clone EA-53, Sigma-Aldrich; 1:200.), anti-Actin, cardiac (clone AC1-20.4.2, Sigma-Aldrich; 1:200), anti-connexin43 (clone CXN-6, Sigma-Aldrich; 1:200), antidesmoplakin (clone DP2.15, Millipore Corporation; 1:200), anti-mMaC myomesin B4 (DSHB; 1:200), and anti-Vinculin (clone hVIN-1, Sigma-Aldrich); rabbit polyclonal antibodies anti-FLAG (F7425, Sigma-Aldrich; 1:200), anti-pan-cadherin (#C3678, Sigma-Aldrich; 1:200); goat polyclonal antibodies anti-FLNC (K-18, Santa Cruz Biotechnology; 1:100); and guinea pig polyclonal antibody anti-HSPB7 (G11W, LTK BioLaboratories; 1:200). After washing in PBS, sections were incubated with secondary antibodies, including FITC- or Rhodamine-conjugated goat anti-mouse and anti-rabbit IgG, FITC- conjugated donkey anti-goat IgG, Rhodamine-conjugated donkey anti-guinea pig IgG secondary antibodies (Jackson ImmunoResearch Laboratories). Fibrosis was detected by staining using Alexa Fluor 647 wheat germ agglutinin (WGA, Invitrogen; 10 μg/ml in PBS) for 10 min at room temperature. Counterstaining was performed using 0.5 μg/ml of Hoechst 33342 (Cell Signaling Technology). Fluorescence was visualized using a Zeiss LSM700 confocal microscope.

### Echocardiography

Two-dimensional echocardiography was performed using a Philips iE33 ultrasound imaging system (Philips Medical Systems, Best, Netherlands) equipped with a 7–15 MHz linear array transducer on 10- to 12-week-old (n = 5 per group) mice. Echocardiography was recorded before tamoxifen administration and at 4 or 7 days after the first tamoxifen treatment for changes in cardiac function. The animals were initially anesthetized using 3% isoflurane. After the animals were sedated, anesthesia was maintained using 1% isoflurane during the echocardiographic examination. Heart rate was maintained between 350 and 600 beats per minute. After two-dimensional long- and short-axis images of the left ventricular (LV) were obtained, M-mode traces were acquired for measurement of the LV chamber dimensions at the diastole and systole, as well as the wall thickness. Echocardiography-derived LV mass, fractional shortening (FS), and ejection fraction were recorded. Measurements were averaged from five consecutive cardiac cycles.

### Surface and telemetric electrocardiogram

Surface ECGs were recorded using a PowerLab 8/30 (AD Instruments, Dunedin, New Zealand). ECGs were used to assess mice before and at 7 days after the first tamoxifen treatment. The animals were anesthetized using 1% isoflurane and standard three-lead surface ECG recordings were performed continuously for 15 minutes. The data were digitized and stored for off-line analysis using LabChart Pro software (AD Instruments).

For telemetric measurement of the ECG, miniature telemetry transmitter devices (HD-X11, Data Sciences International) were implanted subcutaneously on the back with electrodes surgically placed and sutured to the right of the trachea and the left upper abdominal region as described [50]. The animals were allowed 7 days to recover from the surgery before telemetry recordings were acquired. Recordings from control and CKO mice were assessed continuously for 2 hours before and at 4, 7, and 14 days after the first tamoxifen treatment. For CKO mice, recordings were continuous beginning at day 7 after injection until the time of death. The receiver (Physiotel Receiver, model RPC-1; Data Sciences International) was used for data acquisition. ECG signals were digitized and stored for off-line analysis using LabChart Pro software (AD Instruments).

### Transmission electron microscopy

Mouse left ventricle tissue was diced into small blocks in a fixative mixture of glutaraldehyde (1.5%) and paraformaldehyde (1.5%) in phosphate buffer at pH 7.3. The procedure was identical to that described previously [51]. Ultrathin sections were cut, mounted, post-stained, and observed using a FEI TECNAI G2 F20 S-TWIN electron microscope (Electron Microscope Core Facility, Institute of Cellular and Organismic Biology, Academia Sinica).

### Co-immunoprecipitation experiments

For immunoprecipitation experiments, hearts from adult C57BL/6 wild-type and HSPB7Flox/Flox mice were lysed in Cell Lysis Buffer (Cell Signaling Technology) with complete protease inhibitor cocktail (Roche Applied Sciences). Subsequently, 1 mg of total protein extracts were incubated with 50 μL of prewashed anti-FLAG M2 affinity gel (A2220, Sigma-Aldrich) at 4°C overnight. The beads were then washed with PBS and analyzed through western blotting with an anti-FLNC antibody (K-18, Santa Cruz Biotechnology). Wild-type mice heart was used as a negative control. Anti-HSPB7 (G11W, LTK BioLaboratories) was used as a control for the equal loading of heart extracts.

### Evans blue dye injection

For in vivo tests of muscle cell membrane integrity, 8- to 10-week-old control and CKO mice (n = 4 per group) were first treated using 40 mg/kg of tamoxifen (Sigma) through intraperitoneal injection for four consecutive days. Evans Blue dye (EBD; Sigma) with 0.1 mg/g of body weight was intraperitoneally injected for two consecutive days into HSPB7 CKO and control mice 5 days after the first tamoxifen administration. After 18 h, mice were sacrificed and their hearts were harvested and cryosectioned. EBD-positive myofibers were directly observed under a stereomicroscope with blue color (SMZ1500, Nikon) and a fluorescence microscope with red autofluorescence (BX51, Olympus).

### Administration of the recombinant adenovirus

The adenoviruses Ad-Cre (5 × 107 pfu per μL) for Cre recombinase were kindly provided by Guey-Shin Wang (Institute of Biomedical Sciences, Academia Sinica, Taiwan). To achieve successful gene delivery through intracardiac virus infection, 10- to 12-week-old male HSPB7Flox/Flox mice weighing 25–30 g were used in the experiments. Mice were intraperitoneally anesthetized with Avertin at a dose of 100 mg/kg. The skin was incised at the level of the left third and fourth ribs and the pectoral muscles were dissected using two fine forceps and retracted gently with a 6–0 silk suture to free the location. The 27-g needle was inserted 4-mm deep directly into the thorax between the third and fourth ribs. Then, we performed four injections (10 μL each) of Ad-Cre (5 × 10^7^ pfu/μL) into the cardiac wall. After a slow injection, the pectoral muscles were quickly released and the skin was sutured using a 3–0 silk suture, and animals were observed and monitored until recovery. After sacrificing the mice at d8, heart tissue was isolated, directly embedded in OCT, and cut (cryosectioned, 16-μm sections) for further immunofluorescence study.

### Statistics

Results are presented as mean ± s.d. Comparisons between the two groups employed two-tailed Student's t-test. Mouse survival rates were calculated through the Kaplan–Meier method. When analyzing statistical differences between the groups of mice, a P value of less than 0.05 was considered significant.

## Supporting information

S1 Fig Telemetry ECG recordings of HSPB7 CKO mice. Annotated telemetry 2-lead ECG curve of the HSPB7 CKO and control animals at 7 days after tamoxifen administration. A horizontal bar tracing marks the ST segment abnormalities in HSPB7 CKO mice. n = 2 per group.
(TIF)

S2 Fig Expression of sarcomere proteins at control and HSPB7 CKO cardiomyoctes. Confocal micrographs of longitudinal sections of the cardiac muscle of control and CKO mice at d7 after tamoxifen administration. Specific antibodies were used to identify the distributions of sarcomere components: α-actinin (Z-line) and myomesin (M-line). In HSPB7 CKO hearts, the staining revealed the normally striated structure of Z-line and M-line. Insets show representative areas at a higher magnification. The nucleus was visualized through Hoechst 33342 staining. n = 4 per group. Scale bar: 20 μm.
(TIF)

S3 Fig HSPB7 interacts with FLNC protein in vivo. Cardiac muscle from adult HSPB7^Flox/Flox^ or wild-type mice was lysed and incubated with anti-FLAG M2 affinity gel for co-immunoprecipitation (IP) and further immnoblot analysis was conducted to identify the proteins binding to HSPB7. HSPB7 can interact with FLNC and FLNA (A), but not with N-cadherin, desmoplakin, and connexin 43 (B).
(TIF)

S4 Fig HSPB7 colocalizes with FLNC protein in cardiomyocytes. The colocalization of HSPB7 and FLNC was assessed by confocal microscopy. The wild-type heart sections were co-immunostained with antibodies against HSPB7 (red) and FLNC (green). Insets show representative areas at a higher magnification. The nucleus was visualized through Hoechst 33342 staining. Scale bars: 10 μm.
(TIF)

S5 Fig Downregulation of connexin 43 in HSPB7 CKO heart at 4 days after TAM. (A) Immunoblot analysis of intercalated disc-associated proteins and FLNC in HSPB7 CKO and control hearts. GAPDH signal shows the loading of the samples between the lanes. n = 4 per group. (B) Quantitative analysis of immunoblots of protein levels in cardiac tissue from control and CKO mice. Four days after the first tamoxifen injections, only connexin 43 protein expression dropped in CKO animals, as determined by immunoblot analysis. Data are presented as means ± SD. **, *P* < 0.01 relative to the control.
(TIF)

S6 Fig Mislocalization of ID component proteins is not associated with FLNC upregulation and aggregation in HSPB7 CKO cardiomyocytes. Confocal micrographs of longitudinal sections of the cardiac muscle of control and CKO mice at d7 after tamoxifen administration. Antibodies against intercalated disc components desmoplakin (A) or N-cadherin (B), and FLNC are as indicated. In HSPB7 CKO hearts, mislocalization of desmoplakin or N-cadherin (arrowhead) and upregulation of FLNC (arrow) were both observed. Notably, the mislocalization of desmoplakin or N-cadherin (arrowhead) does not always occur with upregulation of FLNC in the same cardiomyocyte (asterisk). The nucleus was visualized through Hoechst 33342 staining. Scale bar: 10 μm.
(TIF)

S7 Fig Disruption of membrane integrity in the HSPB7 CKO heart. For in vivo tests of muscle cell membrane integrity, HSPB7^Flox/Flox^ and CKO mice (n = 4 per group) were first treated with tamoxifen for 4 days, and then injected with EBD at d4 and sacrificed after 18 h. In HSPB7 CKO mice, the heart presented a blue coloration under low-power magnification (5X, left panel) and high-power magnification (40X, middle left panel) compared with the control mice. The pattern of fluorescence microscopy results also showed a high uptake of EBD (red) in the HSPB7 CKO mice under low-power magnification (40X, middle right panel) and high-power magnification (400X, right panel) compared with the control mice.
(TIF)

S8 Fig Gene knockout of HSPB7 in the heart by intramyocardial injection with Adeno-Cre in HSPB7^Flox/Flox^ mouse. Confocal micrographs of the cardiac muscle of HSPB7^Flox/Flox^ mice 8 days after intramyocardial injection with the Adeno-Cre virus. The heart sections were co-immunostained with antibodies against HSPB7 (red) and specific antibodies (green) against ID components desmoplakin, N-cadherin, connexin 43 and FLNC to evaluate their expressions. In HSPB7^Flox/Flox^ hearts, connexin 43 was absent from the intercalated discs, and FLNC expression significantly increased at the HSPB7 depleted region of the Adeno-Cre injected heart (the area above the dotted line). The nucleus was visualized through Hoechst 33342 staining (blue). Scale bar: 50 μm.
(TIF)
