# Supplementary figures and images for: HSPB7 prevents cardiac conduction system defect through maintaining intercalated disc integrity
Source: PLoS Genet. 2017 Aug 21;13(8):e1006984. doi: 10.1371/journal.pgen.1006984 (PMC5587339; doi:10.1371/journal.pgen.1006984)

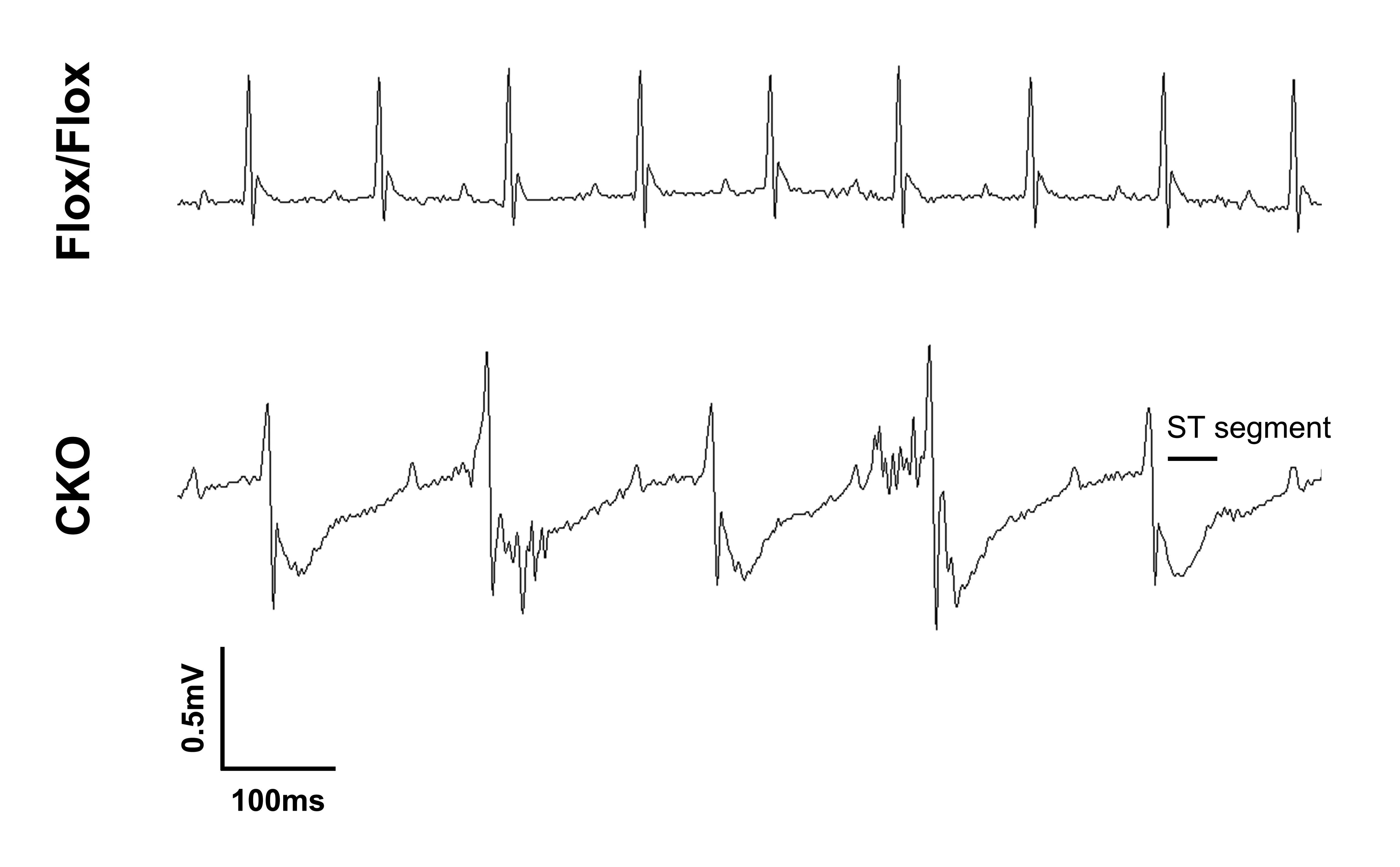

Supplement: S1 Fig — Annotated telemetry 2-lead ECG curve of the HSPB7 CKO and control animals at 7 days after tamoxifen administration. A horizontal bar tracing marks the ST segment abnormalities in HSPB7 CKO mice. n = 2 per group. (TIF) [file pgen.1006984.s001.tif]

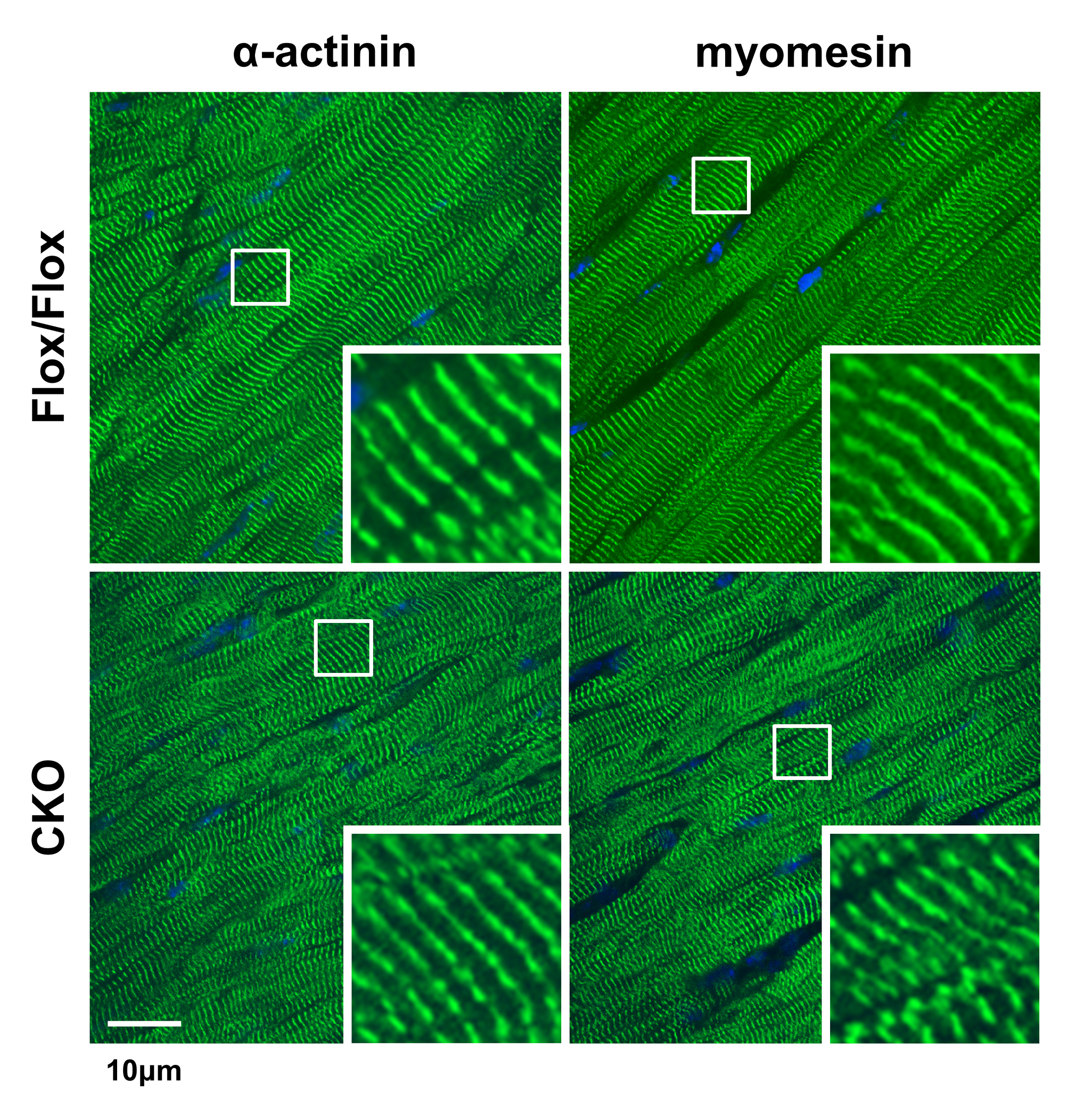

Supplement: S2 Fig — Confocal micrographs of longitudinal sections of the cardiac muscle of control and CKO mice at d7 after tamoxifen administration. Specific antibodies were used to identify the distributions of sarcomere components: α-actinin (Z-line) and myomesin (M-line). In HSPB7 CKO hearts, the staining revealed the normally striated structure of Z-line and M-line. Insets show representative areas at a higher magnification. The nucleus was visualized through Hoechst 33342 staining. n = 4 per group. Scale bar: 20 μm. (TIF) [file pgen.1006984.s002.tif]

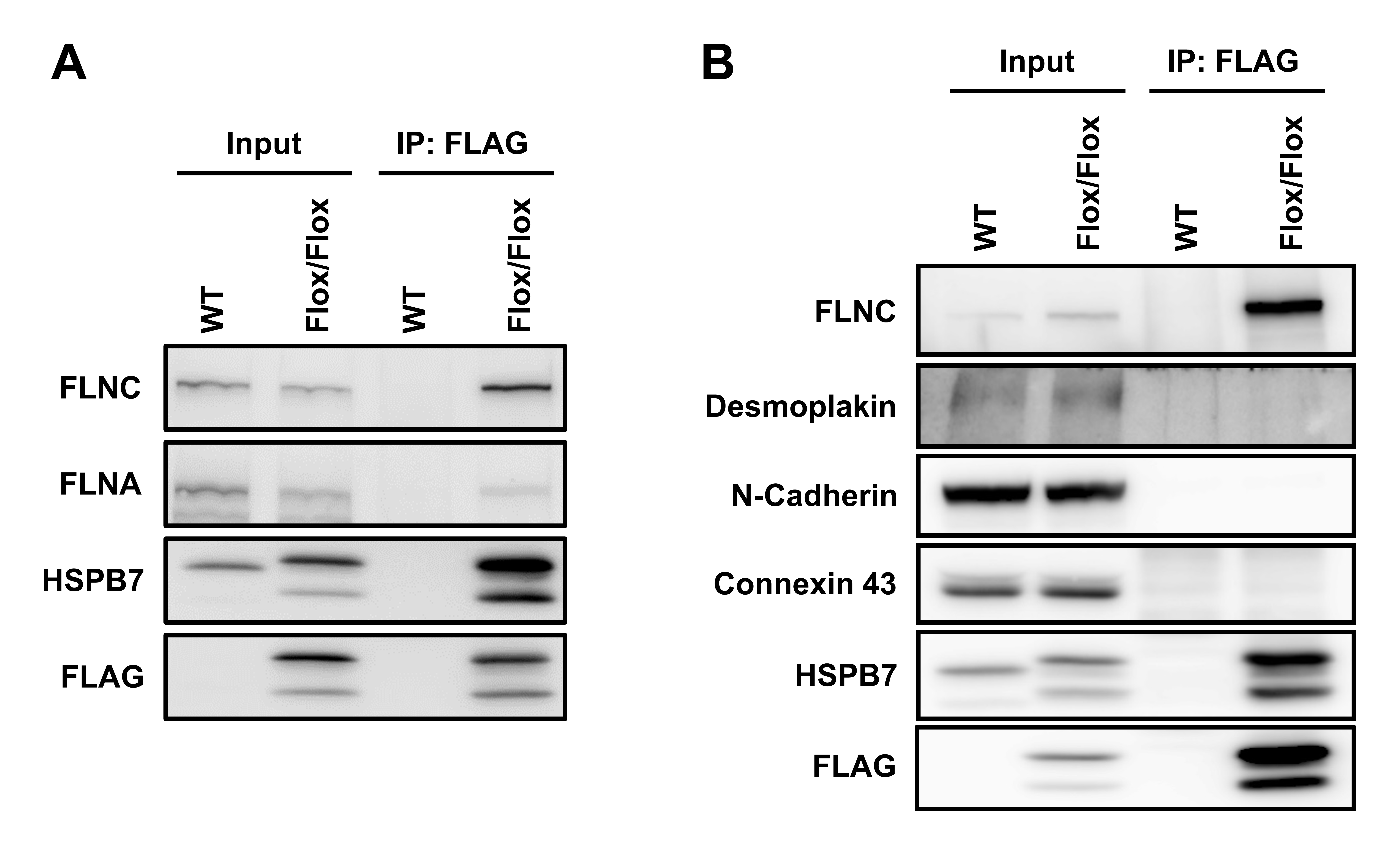

Supplement: S3 Fig — Cardiac muscle from adult HSPB7Flox/Flox or wild-type mice was lysed and incubated with anti-FLAG M2 affinity gel for co-immunoprecipitation (IP) and further immnoblot analysis was conducted to identify the proteins binding to HSPB7. HSPB7 can interact with FLNC and FLNA (A), but not with N-cadherin, desmoplakin, and connexin 43 (B). (TIF) [file pgen.1006984.s003.TIF]

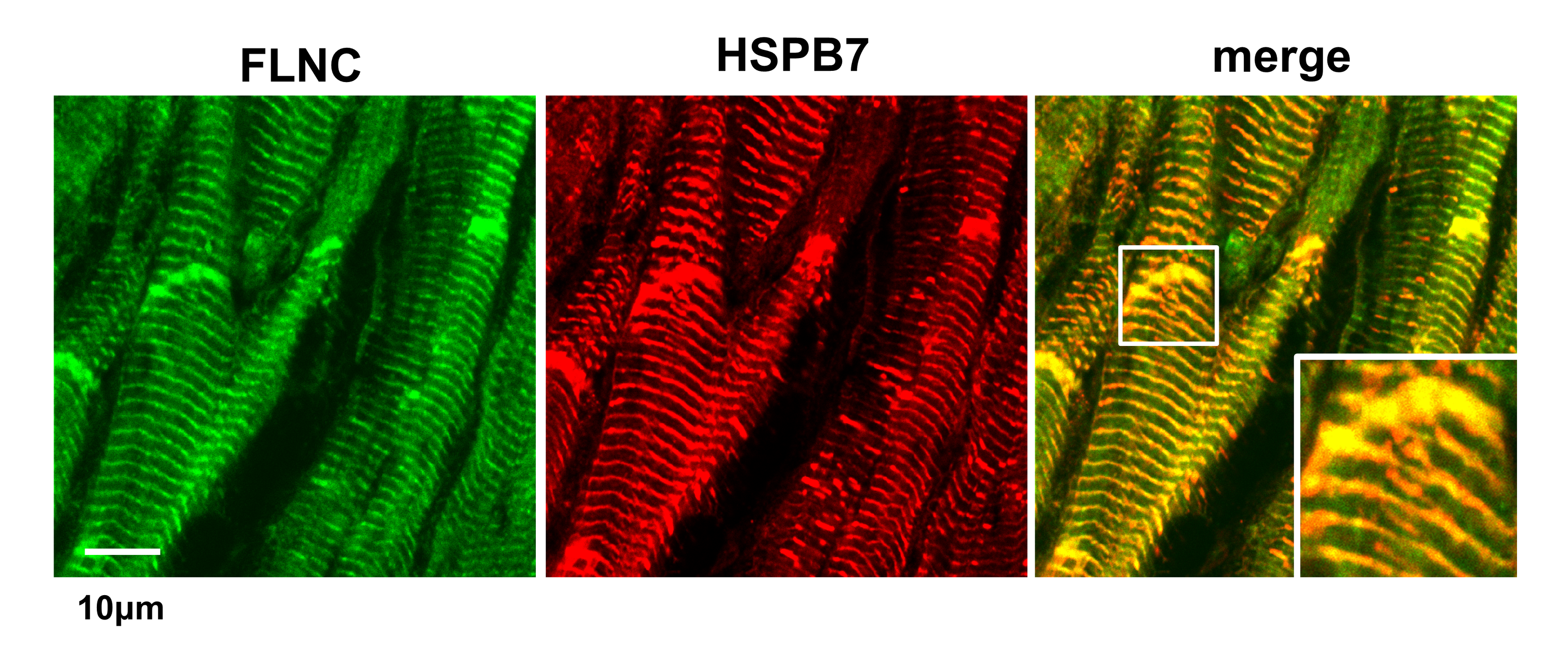

Supplement: S4 Fig — The colocalization of HSPB7 and FLNC was assessed by confocal microscopy. The wild-type heart sections were co-immunostained with antibodies against HSPB7 (red) and FLNC (green). Insets show representative areas at a higher magnification. The nucleus was visualized through Hoechst 33342 staining. Scale bars: 10 μm. (TIF) [file pgen.1006984.s004.tif]

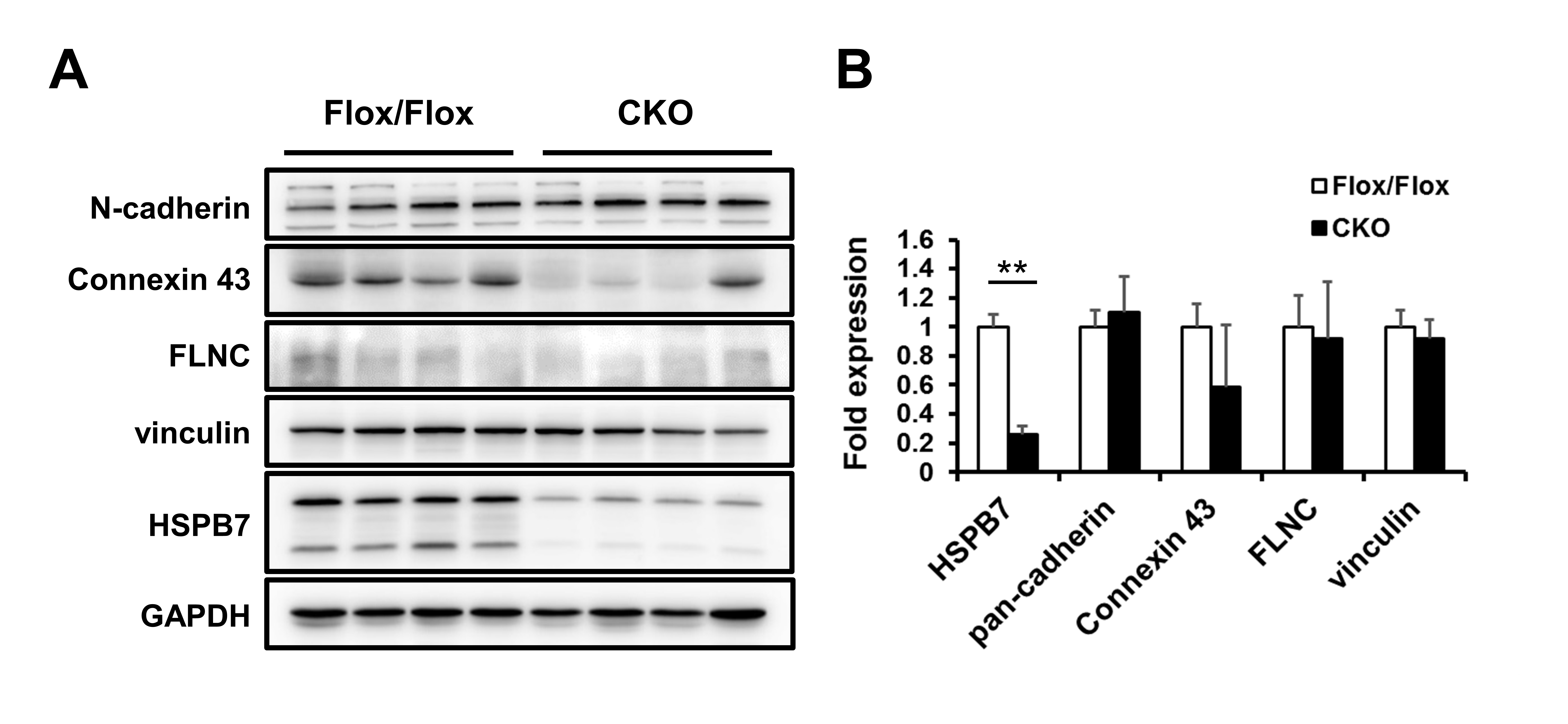

Supplement: S5 Fig — (A) Immunoblot analysis of intercalated disc-associated proteins and FLNC in HSPB7 CKO and control hearts. GAPDH signal shows the loading of the samples between the lanes. n = 4 per group. (B) Quantitative analysis of immunoblots of protein levels in cardiac tissue from control and CKO mice. Four days after the first tamoxifen injections, only connexin 43 protein expression dropped in CKO animals, as determined by immunoblot analysis. Data are presented as means ± SD. **, P < 0.01 relative to the control. (TIF) [file pgen.1006984.s005.TIF]

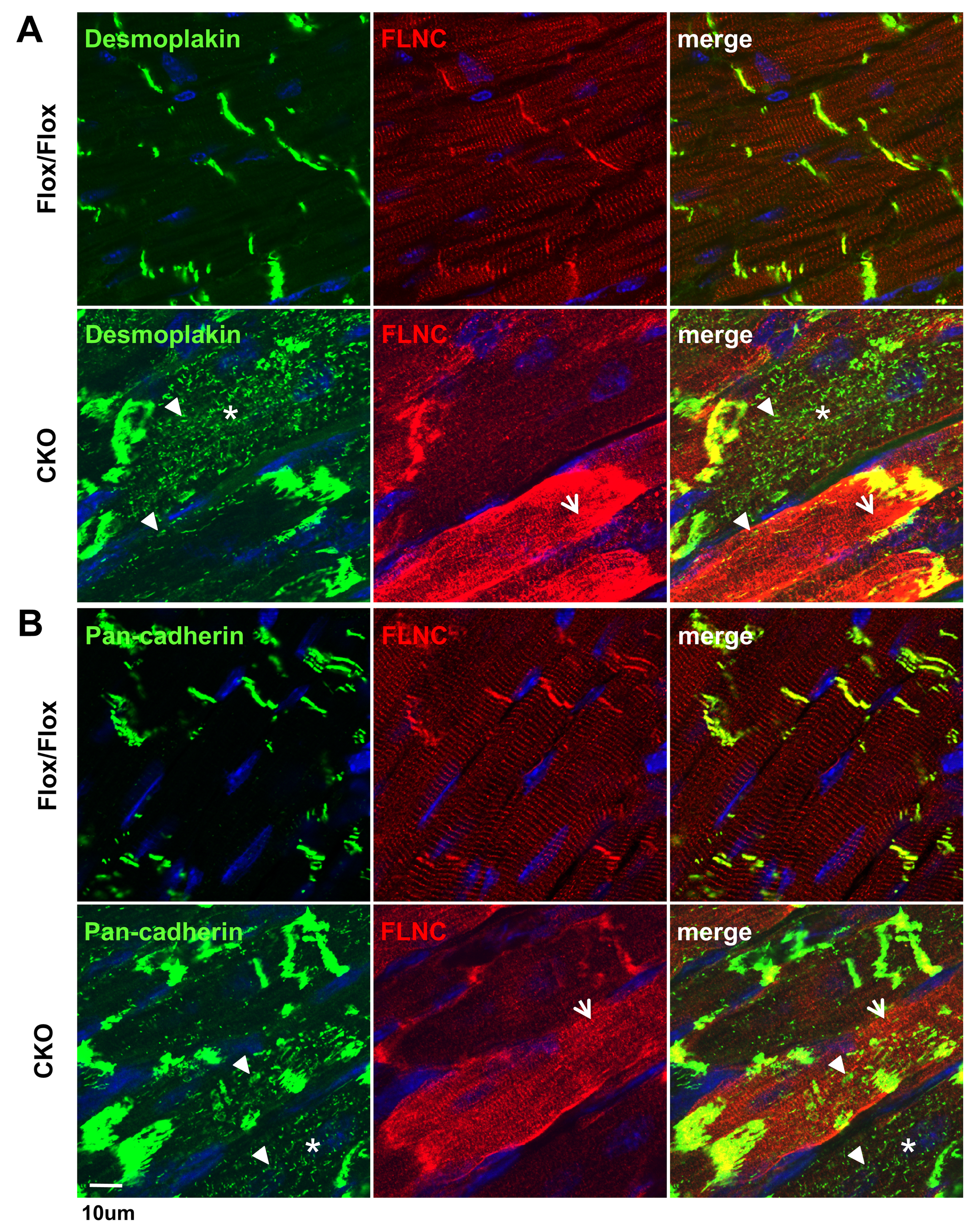

Supplement: S6 Fig — Confocal micrographs of longitudinal sections of the cardiac muscle of control and CKO mice at d7 after tamoxifen administration. Antibodies against intercalated disc components desmoplakin (A) or N-cadherin (B), and FLNC are as indicated. In HSPB7 CKO hearts, mislocalization of desmoplakin or N-cadherin (arrowhead) and upregulation of FLNC (arrow) were both observed. Notably, the mislocalization of desmoplakin or N-cadherin (arrowhead) does not always occur with upregulation of FLNC in the same cardiomyocyte (asterisk). The nucleus was visualized through Hoechst 33342 staining. Scale bar: 10 μm. (TIF) [file pgen.1006984.s006.tif]

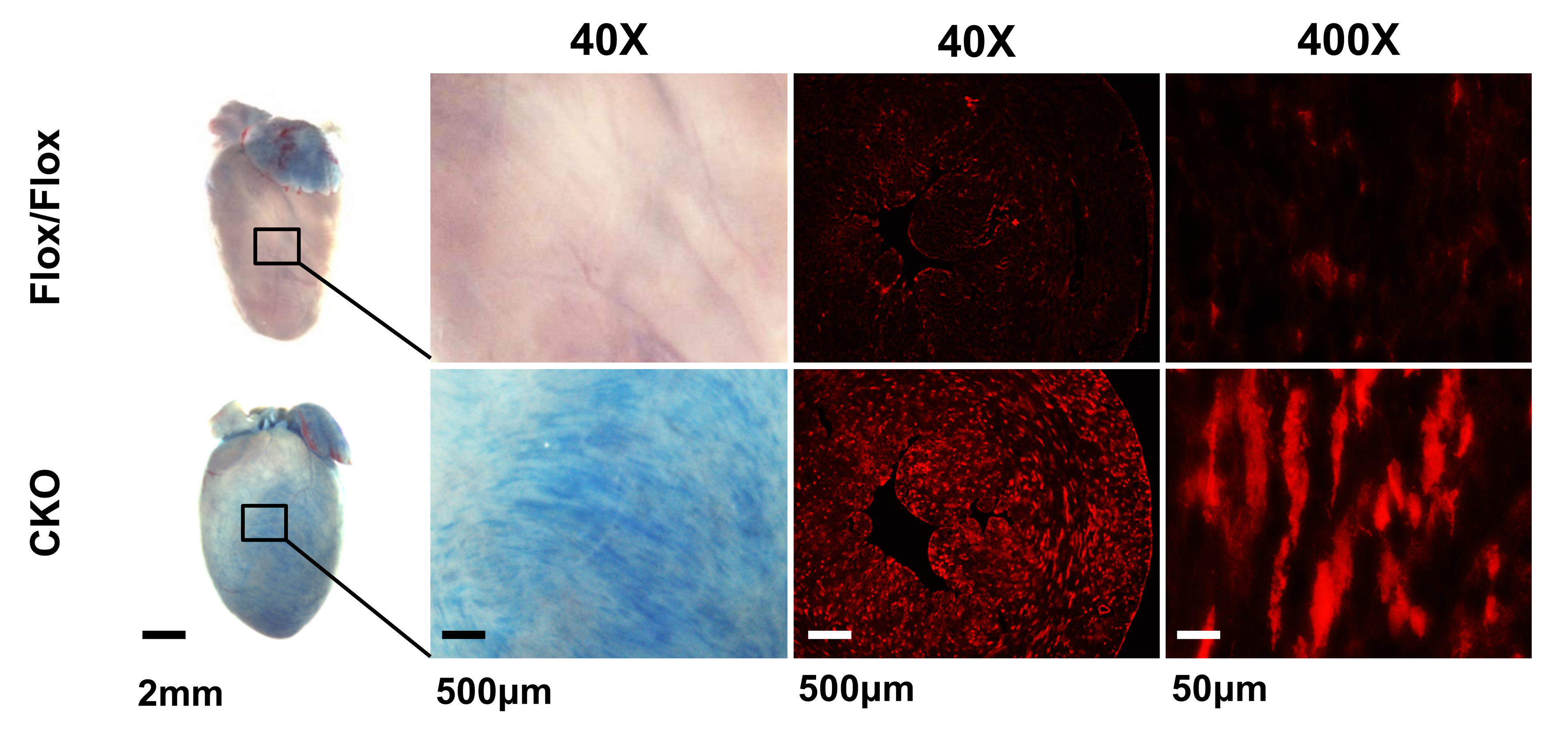

Supplement: S7 Fig — For in vivo tests of muscle cell membrane integrity, HSPB7Flox/Flox and CKO mice (n = 4 per group) were first treated with tamoxifen for 4 days, and then injected with EBD at d4 and sacrificed after 18 h. In HSPB7 CKO mice, the heart presented a blue coloration under low-power magnification (5X, left panel) and high-power magnification (40X, middle left panel) compared with the control mice. The pattern of fluorescence microscopy results also showed a high uptake of EBD (red) in the HSPB7 CKO mice under low-power magnification (40X, middle right panel) and high-power magnification (400X, right panel) compared with the control mice. (TIF) [file pgen.1006984.s007.tif]

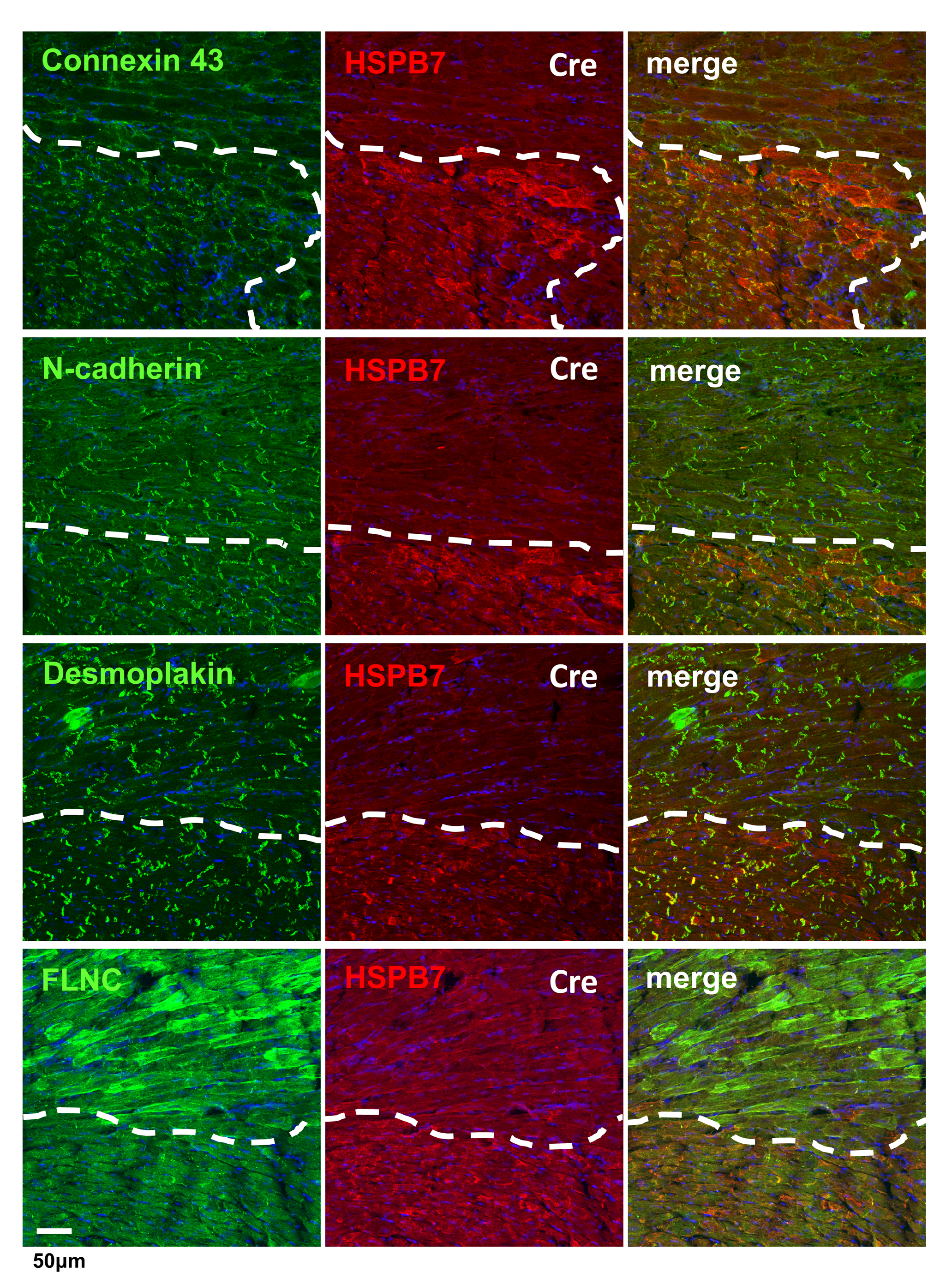

Supplement: S8 Fig — Confocal micrographs of the cardiac muscle of HSPB7Flox/Flox mice 8 days after intramyocardial injection with the Adeno-Cre virus. The heart sections were co-immunostained with antibodies against HSPB7 (red) and specific antibodies (green) against ID components desmoplakin, N-cadherin, connexin 43 and FLNC to evaluate their expressions. In HSPB7Flox/Flox hearts, connexin 43 was absent from the intercalated discs, and FLNC expression significantly increased at the HSPB7 depleted region of the Adeno-Cre injected heart (the area above the dotted line). The nucleus was visualized through Hoechst 33342 staining (blue). Scale bar: 50 μm. (TIF) [file pgen.1006984.s008.tif]
